# A novel protein elicitor (PeSy1) from *Saccharothrix yanglingensis* induces plant resistance and interacts with a receptor‐like cytoplasmic kinase in *Nicotiana benthamiana*

**DOI:** 10.1111/mpp.13312

**Published:** 2023-03-05

**Authors:** Jianxun Wang, Shang Liu, Peng Ren, Fengguo Jia, Feng Kang, Ruolin Wang, Renzheng Xue, Xia Yan, Lili Huang

**Affiliations:** ^1^ College of Life Science Northwest A&F University Yangling China; ^2^ State Key Laboratory of Crop Stress Biology for Arid Areas Northwest A&F University Yangling China; ^3^ College of Plant Protection Northwest A&F University Yangling China

**Keywords:** disease resistance, microbe‐associated molecular pattern, protein elicitor, receptor‐like cytoplasmic kinase, *Saccharothrix yanglingensis*

## Abstract

Previously, we reported a rare actinomycete *Saccharothrix yanglingensis* Hhs.015 with strong biocontrol ability, which can colonize plant tissues and induce resistance, but the key elicitor and immune mechanisms were unclear. In this study, a novel protein elicitor screened from the genome of Hhs.015, PeSy1 (protein elicitor of *S. yanglingensis* 1), could induce a strong hypersensitive response (HR) and resistance in plants. The *PeSy1* gene encodes an 11 kDa protein with 109 amino acids that is conserved in *Saccharothrix* species. PeSy1‐His recombinant protein induced early defence events such as a cellular reactive oxygen species burst, callose deposition, and the activation of defence hormone signalling pathways, which enhanced *Nicotiana benthamiana* resistance to *Sclerotinia sclerotiorum* and *Phytophthora capsici*, and *Solanum lycopersicum* resistance to *Pseudomonas syringae* pv. *tomato* DC3000. Through pull‐down and mass spectrometry, candidate proteins that interacted with PeSy1 were obtained from *N. benthamiana*. We confirmed the interaction between receptor‐like cytoplasmic kinase RSy1 (Response to PeSy1) and PeSy1 using co‐immunoprecipitation, bimolecular fluorescence complementation, and microscale thermophoresis. PeSy1 treatment promoted up‐regulation of marker genes in pattern‐triggered immunity. The cell death it elicited was dependent on the co‐receptors NbBAK1 and NbSOBIR1, suggesting that PeSy1 acts as a microbe‐associated molecular pattern from Hhs.015. Additionally, RSy1 positively regulated PeSy1‐induced plants resistant to *S. sclerotiorum*. In conclusion, our results demonstrated a novel receptor‐like cytoplasmic kinase in the plant perception of microbe‐associated molecular patterns, and the potential of PeSy1 in induced resistance provided a new strategy for biological control of actinomycetes in agricultural diseases.

## Graphical abstract

PeSy1 from *Saccharothrix yanglingensis* activates plant immune responses that enhance resistance against pathogens. RSy1 in *Nicotiana benthamiana* interacts with PeSy1 as a positive immune regulator during this process.

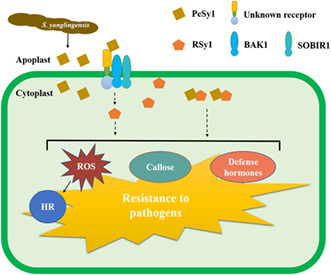

## 1 INTRODUCTION

As the most important producer of the ecosystem, plants have long lived in a complex environment, including a wide range of pathogenic microorganisms, herbivorous insects, and beneficial microorganisms. Some plant pathogens, such as *Sclerotinia sclerotiorum*, *Phytophthora capsici*, and *Pseudomonas syringae* pv. *tomato* (Pst), cause significant economic losses in crop production worldwide (Chen et al., 2020; Granke et al., 2012; Shuang et al., 2006). It is undeniable that fungicides and insecticides are widely used to control diseases and pests to improve crop productivity, but their abuse has caused severe pollution to the environment and other impacts in some cases (Ons et al., 2020; Zaker, 2016). For sustainable agriculture, plant immunity‐based elicitors that induce plant resistance represent a fundamental approach for disease control, due to their environmental friendliness and low cost (Pieterse et al., 2014; Thakur & Sohal, 2013).

To sense and defend against potential pathogens, plants have evolved two different types of immune receptors (Jones & Dangl, 2006). The plasma membrane‐localized pattern recognition receptors (PRRs) recognize the conserved motifs of pathogen‐associated molecular patterns (PAMPs) or microbe‐associated molecular patterns (MAMPs), thereby providing PAMP‐triggered immunity (PTI). Intracellular resistance proteins, usually nucleotide‐binding leucine‐rich repeat‐containing proteins with specific recognition, monitor the presence of pathogen effector proteins resulting in effector‐triggered immunity (ETI) (Bigeard et al., 2015). Recent studies have shown that the boundaries between PTI and ETI are not distinct, and their immune signals overlap and are shared (Ngou et al., 2021; Yuan et al., 2021), such as reactive oxygen species (ROS) outbreaks, callose deposition, defence hormone synthesis, and defence‐related gene expression (Tsuda & Katagiri, 2010; Yu et al., 2017). ROS, including hydrogen peroxide (H_2_O_2_) and superoxide anion ($O_{2}^{\cdot -}$), have dual roles in plant biology (Miller et al., 2010). High concentrations of ROS can lead to the hypersensitive response (HR), while a basal level of ROS is closely related to the regulation of plant growth and hormone signalling (Mittler, 2017; Waszczak et al., 2018). The ROS burst also involves Ca^2+^ and NO signalling, which play a crucial role in plant defence responses, inducing callose deposition on the cell wall to slow down pathogen infection (Nishimura et al., 2003; Shetty et al., 2008). Furthermore, the precise regulation by three defence hormones, salicylic acid (SA), jasmonic acid (JA) and ethylene (ET), plays an important role in the plant fight against systemic spread of pathogens (Spoel & Dong, 2008; Vlot et al., 2021).

Elicitors have steadily emerged as a potent alternative to fungicides and insecticides in green agriculture. Numerous elicitors, such as proteins, sugars, lipids, and peptides, have been isolated and characterized from various pathogens and biocontrol microorganisms (Abdul Malik et al., 2020). PeBL1, the protein elicitor from *Brevibacillus laterosporus* A60, induces a typical HR in tobacco, enhancing plant resistance to tobacco mosaic virus and *P. syringae* pv. *tabaci* (Wang et al., 2015). The protein elicitor OPEL of *Phytophthora parasitica* induces early cellular defence responses and up‐regulation of PTI marker genes and SA pathway genes, and its glycosyl hydrolase (GH) domain is necessary to elicitor activity (Chang et al., 2015). The *S. sclerotiorum* protein elicitor SsCut causes cell death in various plants and improves plant disease resistance by inducing the accumulation of secondary metabolites (Zhang et al., 2014). Despite the diversity of known elicitors, our understanding of how elicitors trigger plant defence responses is still limited (Pršić & Ongena, 2020).

MAMPs), also known as general elicitors, are conserved components of different classes of microorganisms, such as bacterial flagellin, elongation factors, peptidoglycan, and lipopolysaccharides (Abdul Malik et al., 2020; Albert, 2013; Felix et al., 1999; Kunze et al., 2004), which contribute to microbial survival or lifestyle. Plants construct basic immunity by deploying PRRs, including receptor‐like proteins (RLPs) and receptor‐like kinases (RLKs), to sense these MAMP signals (Tang et al., 2017). RLCK is a receptor‐like kinase lacking the extracellular domain in RLK superfamily, which is involved in plant disease resistance, stress resistance, and growing development (Afzal et al., 2008; Liang & Zhou, 2018; Lin et al., 2013). The plasma membrane‐localized cytoplasmic receptor kinase BOTRYTIS‐INDUCED KINASE 1 (BIK1) has been widely studied (Rao et al., 2018). On bacterial flagellin flg22 perception, BIK1 complexes with the RLK flagellin‐sensing 2 (FLS2) and brassinosteroid (BR) insensitive 1‐associated kinase 1 (BAK1), and is phosphorylated as a kinase substrate of BAK1 to transduce flg22 signalling (Bauer et al., 2001; Gómez‐Gómez & Boller, 2000; Lu et al., 2010). Furthermore, BIK1 mediates several MAMP‐triggered responses through interaction with PRRs such as EFR, PEPRs, and CERK1 (Lal et al., 2018; Liu et al., 2013; Zhang et al., 2010). The characterization of novel MAMPs and their corresponding targets not only contributes to the understanding of host–microbe co‐evolution, but also provides a new resource for molecular disease resistance engineering (Abdul Malik et al., 2020; Chisholm et al., 2006).

Actinomycetes are a unique group within the bacterial domain, capable of producing a variety of bioactive substances that have important practical and economic uses, but little research has been done on elicitors. *Saccharothrix yanglingensis* Hhs.015 is an actinomycete isolated from cucumber roots that can effectively control plant pathogens through competition, secretion of antagonistic factors, and induced resistance (Fan et al., 2016; Lu et al., 2018; Wang et al., 2019). Hhs.015 has shown great efficacy against apple Valsa canker, Sclerotinia diseases of rapeseed, and tomato leaf mould in field tests (Chen et al., 2013; Li et al., 2016; Min et al., 2007). Remarkably, Hhs.015 can colonize apple tissue culture seedlings and promote the related defence enzymes activity (Fan et al., 2016; Yan et al., 2019). Protein elicitors have been screened using the whole‐genome data of Hhs.015 (Yan et al., 2019). The protein elicitor BAR11 can induce a defence response in *Arabidopsis thaliana*, and molecular experiments showed that it interacts with catalase in plant cells (Zhang et al., 2018). However, the molecular mechanism of the plant immune response induced by Hhs.015 protein elicitors remains unclear.

Given the ability of Hhs.015 to induce plant resistance, we speculated that there would be a corresponding MAMP recognition mechanism in plants; therefore, we conducted an in‐depth analysis of the genome‐wide map of Hhs.015 to screen for key protein elicitors. In this study, we demonstrated that a novel protein elicitor PeSy1 from Hhs.015 triggered plant defence responses such as the HR, ROS burst, callose accumulation, and resistance against *S. sclerotiorum*, *P. capsici*, and Pst DC3000. PeSy1 acts as a MAMP from Hhs.015 and interacts with the receptor‐like cytoplasmic kinase RSy1, resulting in the PTI response, in *N. benthamiana*. Moreover, RSy1 positively regulates plant immunity and disease resistance. Transient expression of *RSy1* in tobacco showed stronger resistance against *S. sclerotiorum* after PeSy1 treatment. Collectively, this study reveals a novel MAMP PeSy1 in an actinomycete, and the plant RLK family participates in PeSy1‐induced immunity.

## 2 RESULTS

### 2.1 Transient expression of protein elicitor induces cell death in *N. benthamiana*

**FIGURE 1:**
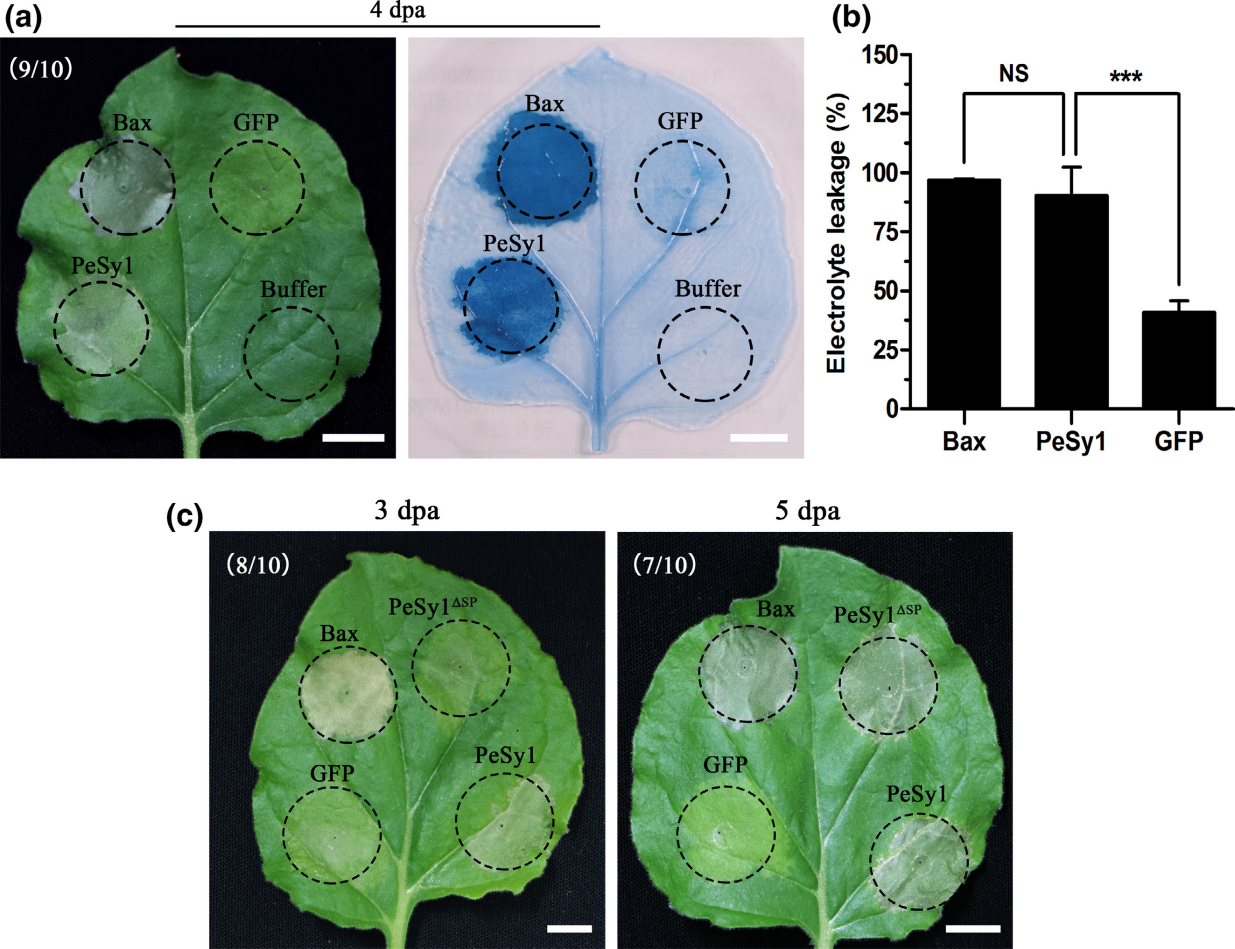
PeSy1 induces cell death in *Nicotiana benthamiana* leaves. (a) Transient expression of PeSy1 in *N. benthamiana* mediated by *Agrobacterium tumefaciens* carrying PVX‐PeSy1. Photographs were taken 4 days postagroinfiltration (dpa). (b) Further observation of necrosis spots by trypan blue staining. (c) The degree of cell death was measured by the electrical conductivity of *N. benthamiana* leaves. (e) Cell death response in *N. benthamiana* expressing PeSy1 and PeSy1^ΔSP^. PVX‐PeSy1 and PVX‐ PeSy1^ΔSP^ were transiently expressed in *N. benthamiana* by agroinfiltration. Photographs were taken 3 and 5 dpa. *Bax* was used a positive control. Green fluorescent protein (GFP) and buffer were used as a negative control. Mean and *SE* were calculated from three biological replicates. The statistical analyses were performed with Student's *t* test. Bars indicate ± *SE*. NS, no significant difference; ***p < 0.001. Bars are 1 cm. SP, signal peptide.

As a form of programmed cell death (PCD), the HR is an important immune component during plant–pathogen interactions (Heath, 2000; Thakur & Sohal, 2013). To search for protein elicitors that induce cell death and are potentially recognized by nonhost plants, 32 genes of Hhs.015 predicted to encode secreted proteins (Table S1) were introduced into *N. benthamiana* by agro‐infiltration. Our results identified five predicted secreted proteins capable of inducing cell death in leaf cells (Figure S1). Transient expression of the secreted protein gene Hhs.015_GM7245 in *N. benthamiana* induced intense cell death after 4 days, and necrotic plaques were clearly observed by trypan blue staining (Figure 1a), therefore we named this protein PeSy1. Previous studies have shown that the degree of cell death is positively correlated with ion leakage (Yu et al., 2012). By measuring the electrolyte permeability of *N. benthamiana* transiently expressing PeSy1, it was found that PeSy1 was significantly different from the negative control green fluorescent protein (*GFP*), but not the positive control *Bax* (Figure 1b).

To further verify the location where PeSy1 works, PeSy1 without its signal peptide (SP) (PeSy1^ΔSP^) was transiently expressed in *N. benthamiana*. The results showed that PeSy1^ΔSP^ also induced cell death at 5 days post‐agroinfiltration (dpa), which was slower than full‐length PeSy1 (Figure 1c). *N. benthamiana* expressing full‐length PeSy began to show cell death symptoms at 3 dpa. These results suggest that PeSy1 may play a role in both intracellular and extracellular processes.

### 2.2 Characterization of PeSy1

**FIGURE 2:**
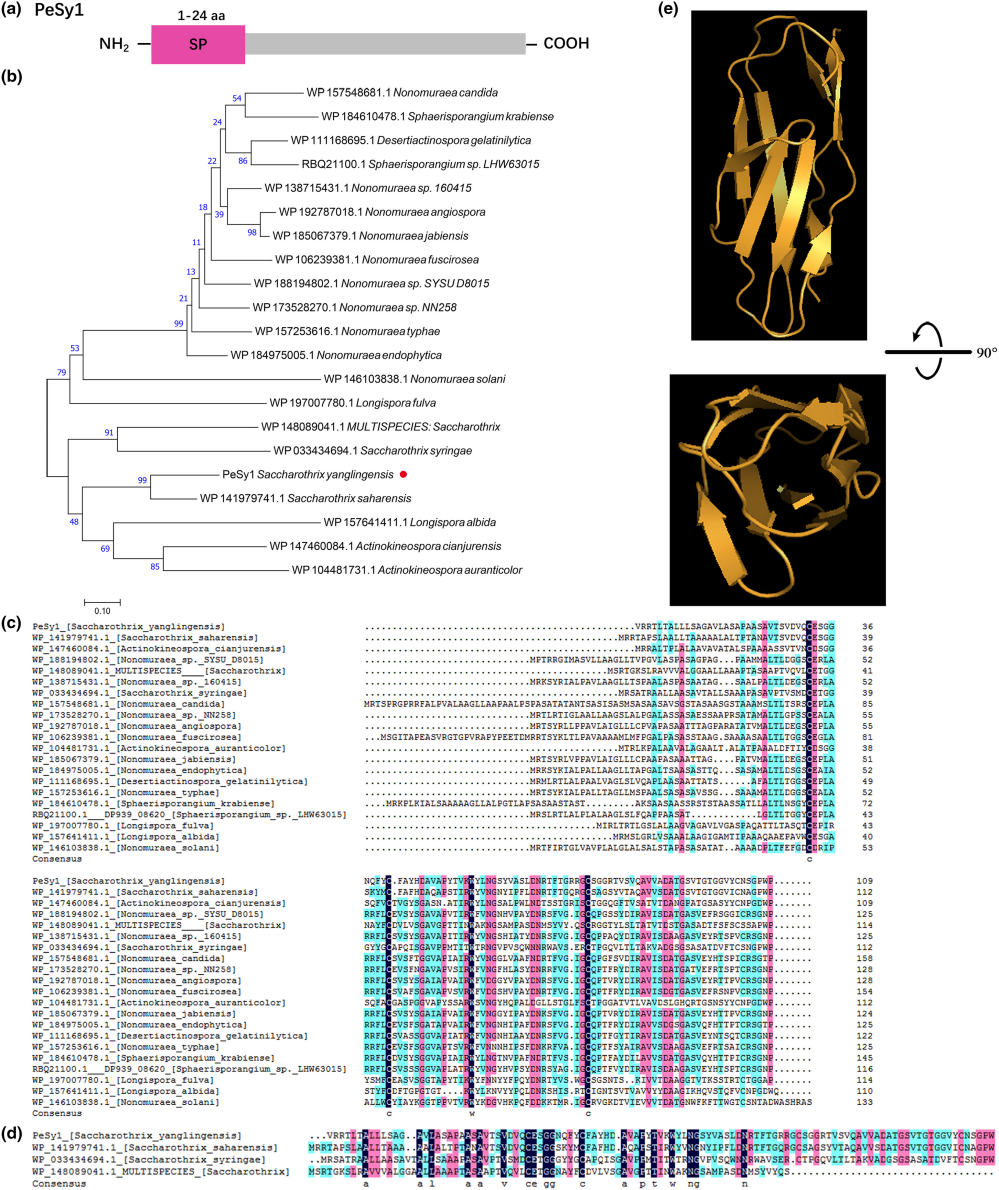
Characterization of PeSy1. (a) Signal peptide (SP) prediction showed that there was an SP of 24 amino acids at the N‐terminal of PeSy1. (b) The phylogenetic tree of PeSy1 protein sequence was constructed based on the neighbour‐joining method. (c, d) Analysis of conserved sites of the PeSy1 sequence. (e) Prediction of PeSy1 tertiary structure.

PeSy1 is a hypothetical protein with no significant structural match other than the SP by SMART analysis (Figure 2a). PeSy1 contains 109 amino acid residues and the first 24 amino acids are the N‐terminal SP. The predicted molecular weight of PeSy1 is 11 kDa and its isoelectric point is 6.70. Twenty sequences with high homology to PeSy1 were obtained from NCBI to construct the phylogenetic tree (Figure 2b). These proteins belonged to hypothetical proteins of rare actinomycetes, which included *Saccharothrix* sp., *Nonomuraea* sp., *Actinokineospora* sp., and *Desertiactinospora* sp. The results showed that PeSy1 belonged to the rare actinomycetes, but the similarity of the protein sequence was only 41.6% (Figure 2c) and it was conserved in *Saccharothrix* sp. with a similarity of 55.9% (Figure 2d). Phyre^2^ Prediction Server showed that the structural confidence of the most matched structure of PeSy1 was 50.4%, which only had nine β‐folds (Figure 2e). These findings indicate that PeSy1 was a novel protein from *S. yanglingensis*.

### 2.3 HR induced by recombinant protein PeSy1‐His in *N. benthamiana*

**FIGURE 3:**
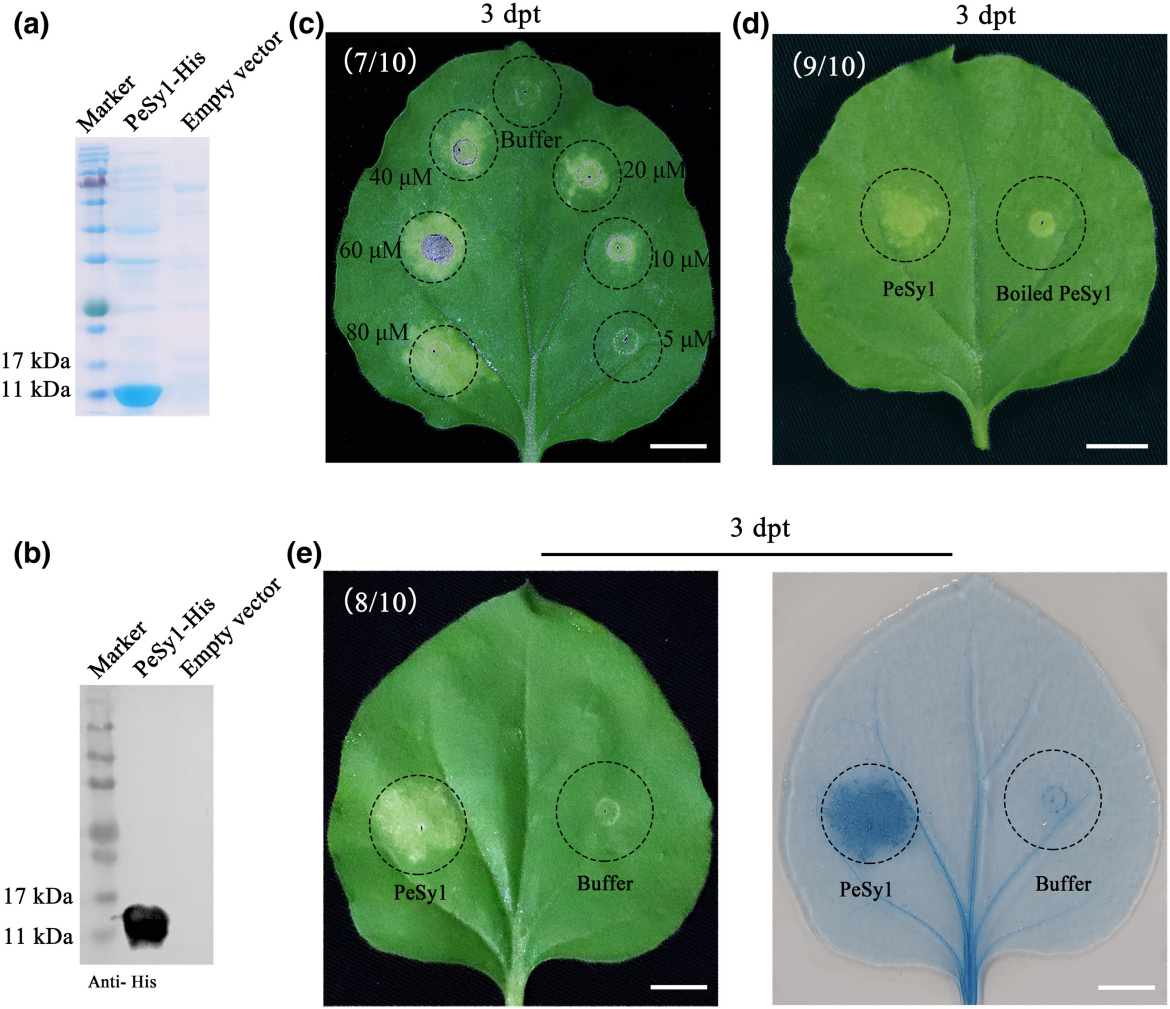
Prokaryotic expression of PeSy1 and induction of hypersensitive response (HR) in *Nicotiana benthamiana* after PeSy1 treatment. (a) Identification of recombinant protein PeSy1‐His by SDS‐PAGE. (b) Verification of PeSy1‐His by western blotting. (c) PeSy1‐His at concentrations of 5, 10, 20, 40, 60, and 80 μM was injected into *N. benthamiana*, then HR was observed and photographed 3 days posttreatment (dpt). (d) 10 μM PeSy1‐His was boiled for 10 min and used to treat tobacco leaves to test the protein thermostability. HR was observed and photographed 3 dpt. (e) Trypan blue staining showing cell death symptoms in 10 μM PeSy1‐His (left) and phosphate‐buffered saline (right) in *N. benthamiana* leaves. Bars are 1 cm.

The recombinant vector pET28a:*PeSy1‐His* was expressed in *Escherichia coli*, and HisPur Ni‐NTA resin was used to purify the recombinant protein. PeSy1‐His protein was detected by SDS‐PAGE (Figure 3a) and it was further verified that the molecular weight was about 11 kDa by western blotting (Figure 3b). *N. benthamiana* leaves were treated with PeSy1‐His at concentrations of 5, 10, 20, 40, 60, and 80 μM. An HR was induced by a minimum of 10 μM at 3 days posttreatment (dpt) (Figure 3c,e). In addition, boiled PeSy1‐His did not cause cell death (Figure 3d). These results indicate that PeSy1‐His induced HR in a dose‐dependent manner.

### 2.4 PeSy1 triggers immunity responses in *N. benthamiana*

**FIGURE 4:**
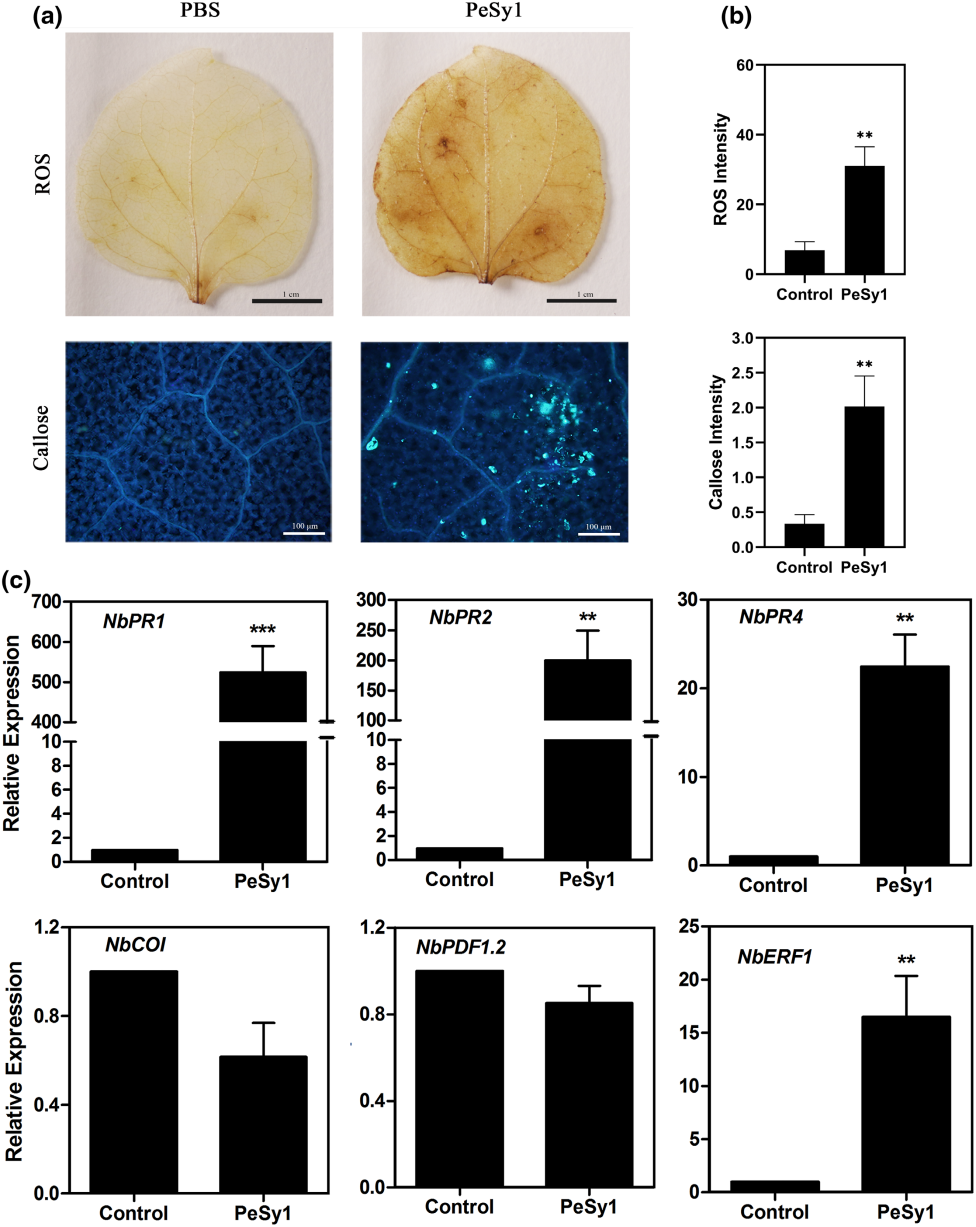
PeSy1 triggers immunity responses in *Nicotiana benthamiana*. (a) *N. benthamiana* leaves were treated with 5 μM PeSy1‐His or buffer control for 24 h. Reactive oxygen species (ROS) were observed as brick red staining after 3,3′‐diaminobenzidine (DAB) reaction (bars are 1 cm) and callose was observed as pale green fluorescence under UV light after aniline blue reaction (bars are 100 μm). (b) Quantification of ROS accumulation and callose deposition in tobacco leaf tissue (*n* = 5) was determined by ImageJ. Mean ± *SE*. (c) *N. benthamiana* leaves were treated with 5 μM PeSy1 or buffer control for 12 h. Relative expression of salicylic acid (*NbPR1*, *NbPR2*) and jasmonic acid (*NbPR4*, *NbCOI* and *NbPDF1.2*) and ethylene (*NbERF1*) pathway‐related marker genes in *N. benthamiana* were determined by reverse transcription‐quantitative PCR analysis. *NbActin* was used as an internal control gene for normalization. Mean and *SE* were calculated from three biological replicates. The statistical analyses were performed with Student's *t* test. Bars indicate ± *SE*. **p < 0.01, ***p < 0.001.

It is already known that HR is accompanied by plant early defence responses, such as ROS burst, callose deposition, and alterations in defence hormone pathways (Han & Hwang, 2017; Schwessinger & Ronald, 2012). To determine whether PeSy1‐induced HR is associated with plant immunity responses, 5 μM PeSy1‐His was injected into *N. benthamiana* leaves. Apparent reddish‐brown ROS accumulation was observed using 3,3′‐diaminobezidine (DAB) staining and pale green fluorescence of callose deposition was observed under UV light excitation after aniline blue staining in leaf cells (Figure 4a,b). As shown in Figure 4c, the relative expression of some genes related to the defence response, such as *NbPR1*, *NbPR2*, *NbPR4* and *NbERF1*, after PeSy1 treatment significantly increased compared with the control. *NbPR1* and *NbPR2* are marker genes for SA‐dependent immunity, and *NbPR4* and *NbERF1* are marker genes for JA/ET‐dependent immunity. Thus, PeSy1 may function as a protein elicitor and induce plant defence responses mediated by SA‐ and JA/ET‐dependent signalling pathways.

### 2.5 PeSy1 enhances plant resistance to pathogens

**FIGURE 5:**
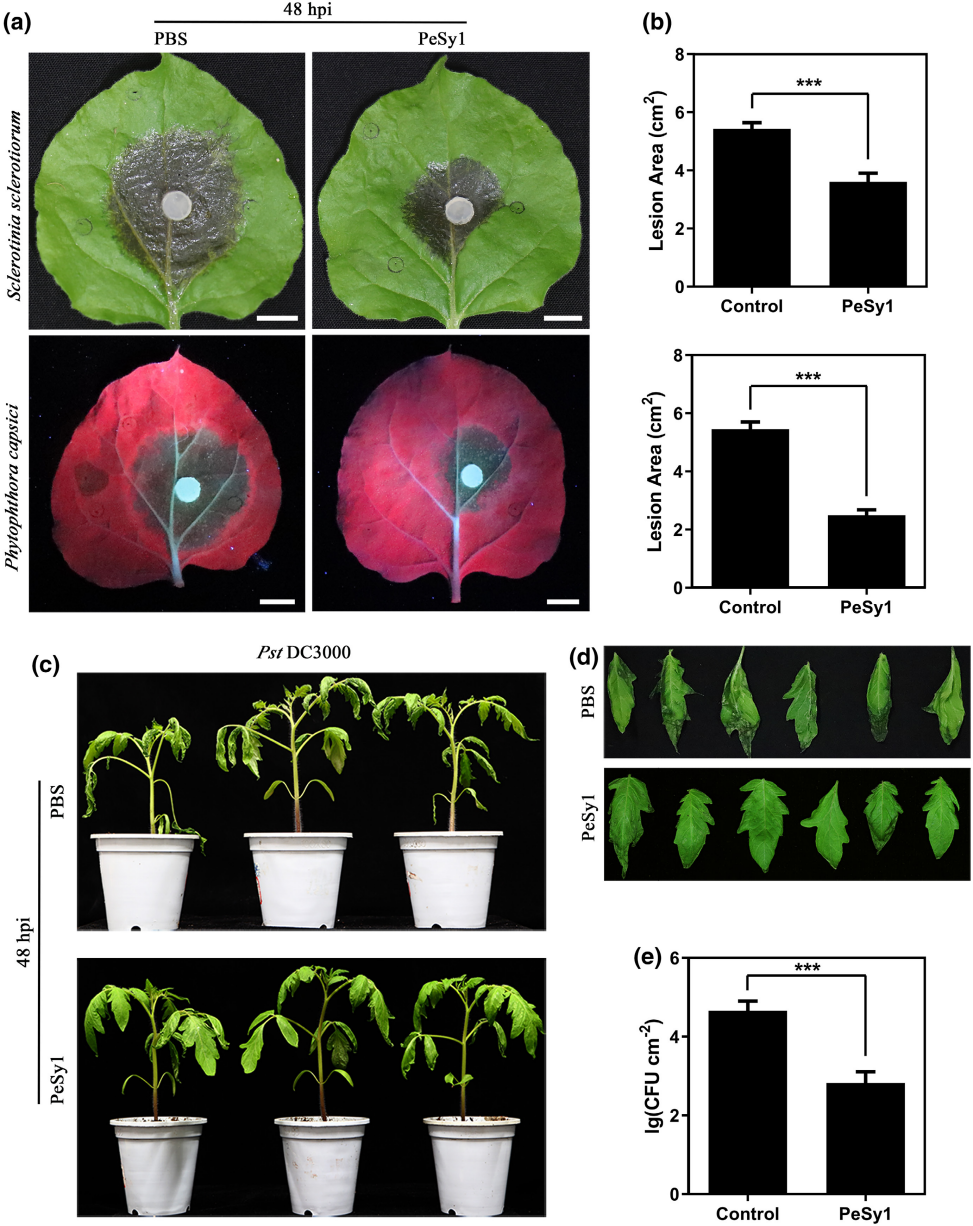
Resistance of plants to pathogens after PeSy1 treatment. (a, b) Representative leaves showing *Sclerotinia sclerotiorum* and *Phytophthora capsici* infection after treatment with 5 μM PeSy1‐His or phosphate‐buffered saline (PBS) control. At 12 h posttreatment (hpt) the *Nicotiana benthamiana* leaves (*n* = 9) were inoculated by *S. sclerotiorum* or *P. capsici*. The lesion area at 48 h postinoculation (hpi) decreased 33.5% and 54.2%, respectively. Photographs were taken 48 hpi. Bars are 1 cm. (c–e) Plants were sprayed with 10 μM PeSy1‐His or buffer control. After 24 h, tomato leaves (*n* = 9) were challenged by *Pseudomonas syringae* pv. *tomato* DC3000 and photographed 48 hpi. Compared with the control‐treated leaves, the density of bacteria in PeSy1‐treated leaves was significantly reduced. Bars are 1 cm. The experiment was conducted three times with similar results. The statistical analyses were performed with Student's *t* test. Bars indicate ± *SE*. ***p < 0.001.

*N. benthamiana* leaves treated with 5 μM PeSy1‐His or phosphate‐buffered saline (PBS) control for 12 h were inoculated with *S. sclerotiorum* or *P. capsici*. As shown in Figure 5a,b, the lesion area of *N. benthamiana* leaves (*n* = 9) reduced by 33.5% and 54.2%, respectively, 48 h postnoculation (hpi) (Figure 5a,b). To verify whether PeSy1 enhances systemic resistance in different plants, experiments were carried out using tomato (*Solanum lycopersicum*) seedlings. The tomato leaves were uniformly sprayed with 10 μM PeSy1‐His or PBS control 24 h before Pst DC3000 inoculation. We found that PeSy1 treatment activated the immune response of tomato seedlings, reduced DC3000 infection, and decreased the pathogen density in tomato leaves (*n* = 9) 48 hpi significantly (Figure 5c–e). These results reveal that PeSy1 can induce broad‐spectrum resistance against various pathogens in plants.

### 2.6 PeSy1 interacts with a receptor‐like cytoplasmic kinase in *N. benthamiana*

**FIGURE 6:**
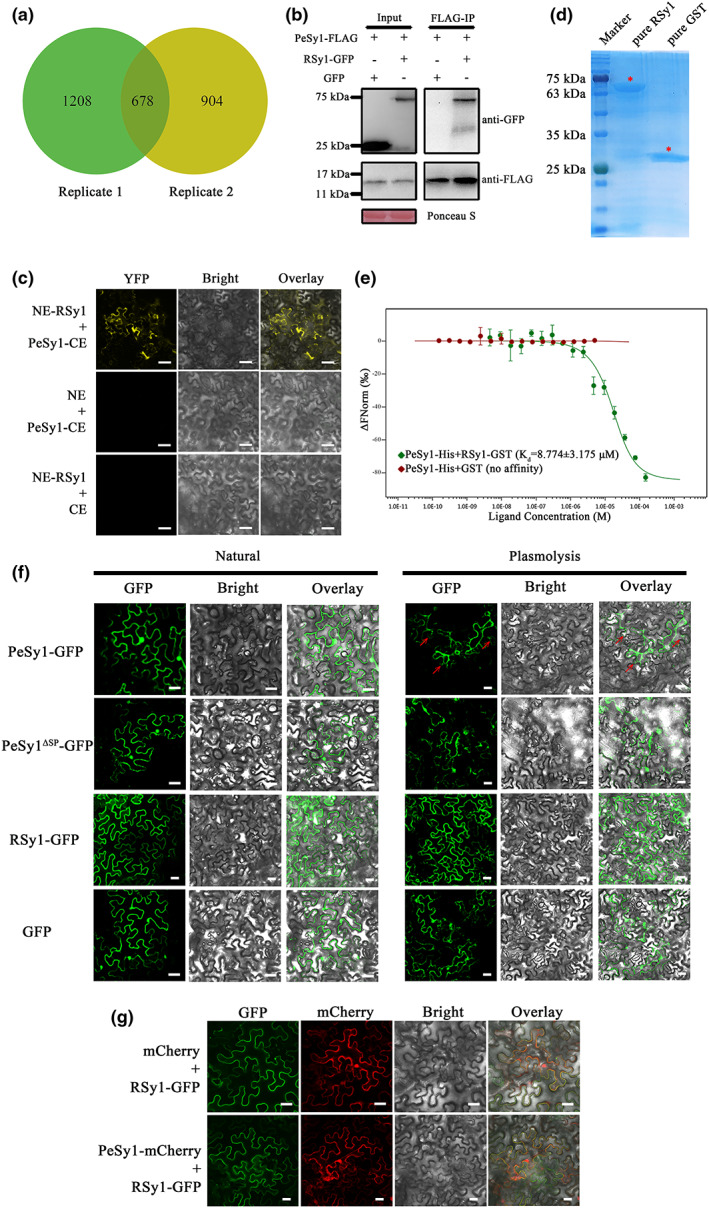
PeSy1 interacts with RSy1. (a) Venn diagram illustrating the number of proteins identified in the mass spectrometry results. (b) The interaction between PeSy1 and RSy1 was detected by co‐immunoprecipitation. Ponceau S (bottom) was used as a loading control. (c) Bimolecular fluorescence complementation showed that PeSy1 interacted with RSy1 in the leaf cells of *Nicotiana benthamiana*. NE‐PeSy1 and RSy1‐CE were co‐agroinfiltrated into *N. benthamiana* leaves. Yellow fluorescence images were taken 48 h postagroinfiltration (hpa). (d) RSy1‐glutathione‐S‐transferase (GST) GST recombinant protein was obtained by prokaryotic expression. The red asterisks indicate the positions of RSy1‐GST and GST proteins. (e) The combination of PeSy1 and RSy1 was detected by microscale thermophoresis (MST). The dissociation constant (*K*_d_) was obtained by fitting with different concentrations of target as abscissa and the changes in fluorescence of labelled PeSy1 as ordinate. GST was used as a reference control. Values represent means ± *SE* from three biological repeats. (f) Subcellular localization of PeSy1 and RSy1. pCAMBIA1302 constructs were transfected into *N. benthamiana* leaves. Transient expression of green fluorescent protein (GFP)‐tagged PeSy1, PeSy1 without signal peptide (PeSy1^ΔSP^), RSy1 and GFP control. Arrows point to the fluorescence of PeSy1 in apoplasts after plasmolysis. (g) Colocalization of PeSy1 and RSy1 in *N. benthamiana*. pICH86988‐PeSy1‐mCherry and pCAMBIA1302‐RSy1‐GFP were co‐agroinfiltrated into *N. benthamiana* leaves. Photographs were taken 2 days postagroinfiltration. Bars are 30 μm.

Protein elicitors bind to the target proteins in plants and cause the plant immune response. To further explore how PeSy1 exerts its elicitor activity, the proteins pulled down by PeSy1‐His in *N. benthamiana* were analysed using liquid chromatography–tandem mass spectrometry (LC–MS/MS). A total of 678 proteins were identified in each of two independent mass spectrometry tests (Figure 6a). Considering that PeSy1 may play a role in both intracellular and extracellular spaces, 14 candidate targets were selected to identify their interaction relationships with PeSy1 (Table 1). The interaction of PeSy1 with several candidate targets was tested using a co‐immunoprecipitation (Co‐IP) assay (Figure S2). One of these, a receptor‐like protein kinase (Niben101Scf02819g03010.1), was designated as RSy1 (response to PeSy1). PeSy1‐FLAG and RSy1‐GFP were co‐expressed in *N. benthamiana* leaves using green fluorescent protein (GFP) as a control, and a Co‐IP assay in vivo was performed using anti‐FLAG magnetic beads. The results showed that all genes were successfully expressed in *N. benthamiana* leaves and detected in the total protein extract. Immunoblot analysis indicated that RSy1‐GFP was detected by the anti‐GFP antibody in the PeSy1‐FLAG‐precipitated immune complex (Figure 6b). To further confirm their interaction, we performed a bimolecular fluorescence complementation (BiFC) assay in *N. benthamiana*. As shown in Figure 6c, PeSy1 interacted with RSy1 in vivo.

**TABLE 1:** Selected candidate proteins that interacted with PeSy1 in pull‐downs from *Nicotiana benthamiana.*

Accession	Description	Number of peptides
Niben101Ctg10591g00003.1	Catalase	34
Niben101Scf09075g03002.1	Xyloglucan endotransglucosylase/hydrolase 6	11
Niben101Scf07619g00006.1	Receptor‐like protein kinase	3
Niben101Scf01764g03022.1	1‐deoxy‐d‐xylulose 5‐phosphate reductoisomerase	19
Niben101Scf11721g00002.1	Receptor kinase 3	3
Niben101Scf10834g03005.1 sp	Calreticulin‐3	1
Niben101Scf02461g00004.1	Phosphoglycerate kinase family protein	18
Niben101Scf01269g08008.1	Calcium‐dependent protein kinase 33	3
Niben101Scf00597g03003.1 sp	26S protease regulatory subunit 8	5
Niben101Scf02819g03010.1	Receptor‐like protein kinase	1
Niben101Scf10910g00006.1	Phosphoribulokinase/uridine kinase family	7
Niben101Scf05688g08010.1	Phosphoglycerate kinase family protein	16
Niben101Scf04386g04007.1	Leucine‐rich repeat receptor‐like protein kinase family protein	3
Niben101Scf06998g02002.1 sp	Subtilisin‐like protease	5

A microscale thermophoresis (MST) assay was performed to investigate the binding capacity of the PeSy1–RSy1 association in vitro. RSy1‐GST protein was obtained by prokaryotic expression using the pGEX‐6p‐1 vector and concentrated to 150 μM using ultrafiltration tubes with appropriate molecular mass (Millipore) (Figure 6d). The MST results showed that PeSy1‐His and RSy1‐GST could form a saturated S‐shaped binding curve with a dissociation constant (*K*_d_) of 8.77 ± 3.18 μM and signal‐to‐noise ratio of 19.29 (Figure 6e). On the contrary, no binding curve was formed between PeSy1‐His and GST. These data suggest that PeSy1 and RSy1 can bind with high affinity in vitro.

RSy1 contains 365 amino acid residues with a molecular weight of 40.95 kDa and an isoelectric point of 6.09. Analysis of functional domains showed that RSy1 contains only the serine/threonine kinase domain (Figure S3), suggesting that RSy1 belongs to the typical RLCK family (Liang & Zhou, 2018). Further analysis of subcellular localization revealed that PeSy1 was localized in the plasma membrane and nucleus, and RSy1 was localized in the cytoplasmic membrane and cytoplasm under natural conditions (Figure 6f), while PeSy1 carrying its signal peptide was observed in the apoplast under plasmolysis conditions, which indicates that the signal peptide of PeSy1 may have an exocrine function (Figure 6f). In addition, considering the interaction between PeSy1 and RSy1, we used PeSy1‐mcherry and RSy1‐GFP to determine the colocation of PeSy1 and RSy1. As shown in Figure 6g, the red fluorescence of PeSy1‐mCherry substantially overlapped with the green fluorescence of RSy1‐GFP in the plasma membrane and cytoplasm. These results prove that PeSy1 could function in the apoplast and interact with RSy1 in the plasma membrane and cytoplasm.

### 2.7 PeSy1 is considered as a MAMP

**FIGURE 7:**
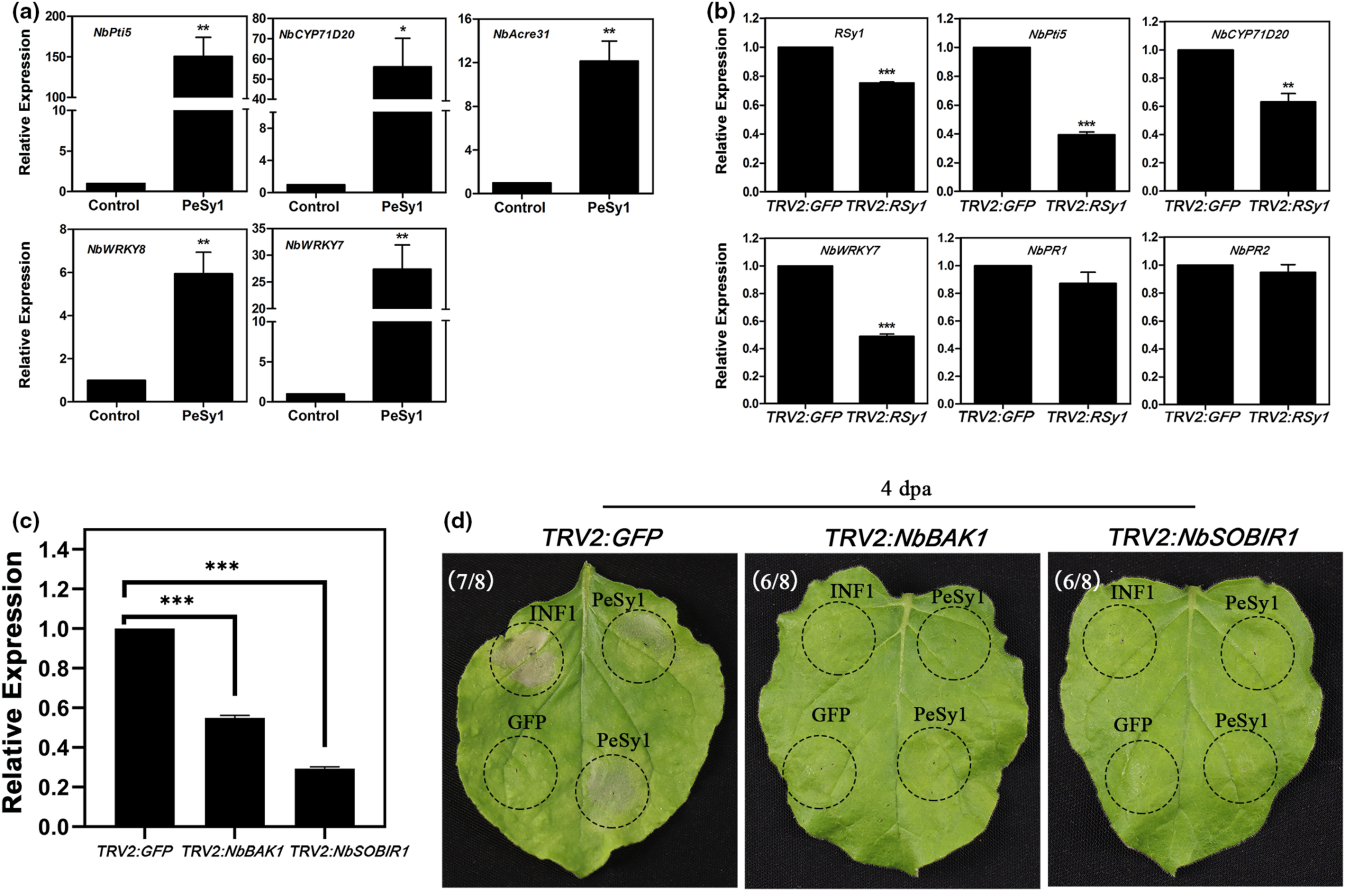
PeSy1 acts as a microbe‐associated molecular pattern (MAMP) from Hhs.015. (a) Transcript accumulation of PAMP‐triggered immunity (PTI) marker genes in *Nicotiana benthamiana* was determined after PeSy1 treatment by reverse transcription‐quantitative PCR (RT‐qPCR) analysis. The *N. benthamiana* leaves were treated with 5 μM PeSy1 or buffer control for 12 h. Expression levels of *NbPti5*, *NbCYP71D20*, *NbAcre31*, *NbWRKY8*, and *NbWRKY7* are shown as fold changes in relation with the control. (b) The expression of *RSy1* and PTI marker genes in *RSy1‐*silenced plants was analysed by RT‐qPCR. The *RSy1*‐silenced *N. benthamiana* was treated with 5 μM PeSy1 for 12 h, and the expression levels of *NbPti5*, *NbCYP71D20*, *NbWRKY7*, *NbPR1*, and *NbPR2* were measured. (c) *NbBAK1* and *NbSOBIR1* expression levels after virus‐induced gene silencing treatment determined by RT‐qPCR analysis. (d) PeSy1 and INF1 induce cell death responses in *NbBAK1‐* and *NbSOBIR1‐*silenced *N. benthamiana*. *Agrobacterium tumefaciens* carrying the PVX constructs was infiltrated into gene‐silenced plants. Photographs were taken 4 days postagroinfiltration (dpa). The *GFP*‐silenced plants were used as the control group. *NbActin* was used as the internal reference gene to standardize the samples. Mean and *SE* were calculated from three biological replicates. The statistical analyses were performed with Student's *t* test. *p < 0.05, **p < 0.01, ***p < 0.001.

According to the above results, PeSy1 was conserved in *Saccharothrix* sp. and recognized by an RLK family protein of *N. benthamiana*. Thus, we speculate that PeSy1 could act as a MAMP from Hhs.015. The expression of PTI marker genes in *N. benthamiana* after PeSy1 treatment was examined by reverse transcription‐quantitative PCR (RT‐qPCR). These results show that 5 μM PeSy1 significantly up‐regulated the expression of the PTI marker genes *NbPti5*, *NbCYP71D20*, *NbAcre31*, *NbWRKY8*, and *NbWRKY7* (Figure 7a), indicating that PeSy1 is likely to be a MAMP.

To verify that RSy1 is involved in PTI signal transduction induced by PeSy1, the expression of *RSy1* was detected by RT‐qPCR after silencing *RSy1* in *N. benthamiana* for 3 weeks. As shown in Figure 7b,c, the expression of RSy1 was significantly down‐regulated, indicating that the *RSy1*‐silenced *N. benthamiana* was successfully obtained. Afterwards, 5 μM PeSy1‐His was injected into the silenced leaves, and RNA was extracted 12 h later to measure the expression of PTI marker genes and *PR* genes. Compared with the controls, transcription of PTI marker genes *NbPti5*, *NbCYP71D20*, and *NbWRKY7* was impaired, but *PR*‐related genes were not markedly changed in *RSy1*‐silenced plants.

One of the features of a MAMP is dependence on co‐receptors. Research has shown that NbBAK1 and NbSOBIR1 interact with most PRRs to facilitate intracellular signalling and have been shown to be critical for PTI (Heese et al., 2007; Liebrand et al., 2014). To test whether PeSy1‐induced cell death is associated with co‐receptors, virus‐induced gene silencing (VIGS) constructs were used to target *NbBAK1* and *NbSOBIR1* expression in *N. benthamiana*. Three weeks after agroinfiltration of TRV constructs, the expression levels of *NbBAK1* and *NbSOBIR1* were significantly decreased in the corresponding plants using RT‐qPCR (Figure 7c). *Agrobacterium tumefaciens* carrying the constructs PVX‐PeSy1 or positive control PVX‐INF1 were infiltrated into gene‐silenced *N. benthamiana* leaves using PVX‐GFP as a negative control. This showed that PeSy1 and INF1 failed to trigger cell death in both *NbBAK1‐* and *NbSOBIR1‐*silenced plants, but not in plants treated with TRV2:*GFP* (Figure 7d). These findings further support that PeSy1 act as a MAMP, and its elicited cell death is dependent on the co‐receptors NbBAK1 and NbSOBIR1.

### 2.8 RSy1 positively regulates *S. sclerotiorum* resistance after PeSy1 treatment

**FIGURE 8:**
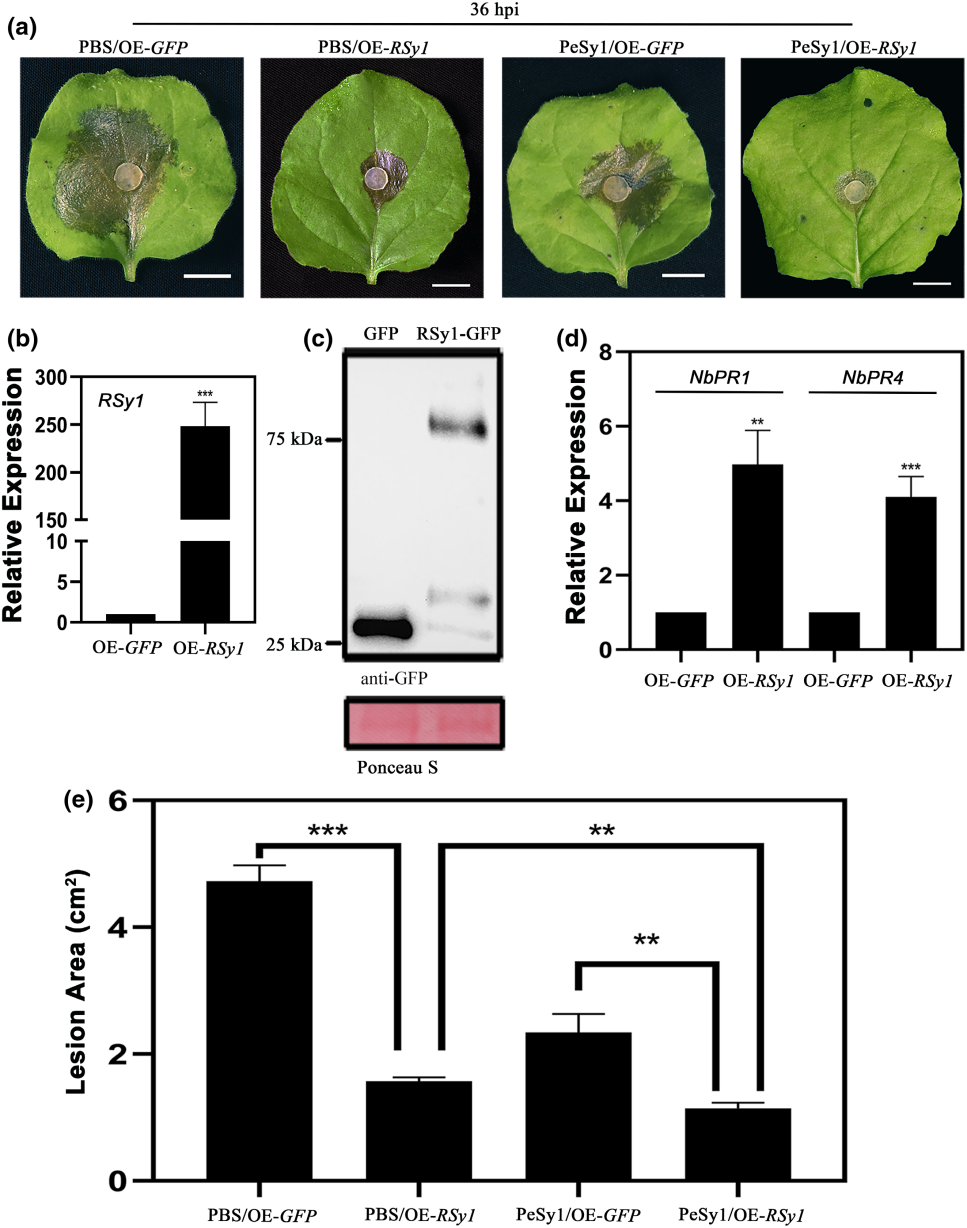
RSy1 as an immune regulator contributes to PeSy1‐induced *Nicotiana benthamiana* resistance against *Sclerotinia sclerotiorum*. (a) Representative leaves showing disease lesions of *GFP‐* or *RSy1‐*overexpressing *N. benthamiana* infected with *S. sclerotiorum*. RSy1 with green fluorescent protein (GFP) tag was transiently expressed in *N. benthamiana* by agroinfiltration, and the leaves (*n* = 5) treated with 5 μM PeSy1‐His or phosphate‐buffered saline (PBS) control were inoculated with *S. sclerotiorum*. Photographs were taken 36 h postinoculation (hpi). Bars are 1 cm. (e) Lesion area was measured 36 hpi. (b) The *RSy1* expression levels in overexpressing (OE) plants were analysed by reverse transcription‐quantitative PCR. (c) An anti‐GFP antibody was used to analyse the expression of RSy1‐GFP fusion protein. (d) Transcript accumulation of *NbPR1* and *NbPR4* in OE‐*RSy1* plants. *NbActin* was used as the internal reference gene to standardize the samples. The OE‐*GFP* was used as the control group. Bars indicate *± SE*. The statistical analyses were performed with Student's *t* test. **p < 0.01, ***p < 0.001. These experiments were repeated three times with similar results.

Because silencing of RSy1 resulted in impaired PeSy1‐induced MAMP signalling, we speculated that RSy1 might affect PeSy1‐induced plant resistance to pathogens. *Agrobacterium*‐mediated transient expression in *N. benthamiana* was used to investigate the function of RSy1. The results of RT‐qPCR indicated that *RSy1* was up‐regulated in *RSy1*‐overexpressing (OE) tobacco by about 248‐fold, compared with *GFP*‐overexpressing controls (Figure 8b). The results of immunoblot analysis showed the successful expression of RSy1‐GFP fusion protein in *RSy1*‐overexpressing plants (Figure 8c). A *S. sclerotiorum* inoculation assay showed that the lesion area of OE‐*RSy1* tobacco was reduced by about 67%, compared with OE‐*GFP* tobacco treated with PBS. Tobacco resistance conferred by *RSy1* overexpression was enhanced after PeSy1 treatment (Figure 8a,e). Moreover, transcriptional analysis of defence‐related genes revealed that *NbPR1* and *NbPR4* were significantly up‐regulated in OE‐*RSy1* plants (Figure 8d). Taken together, these results show that RSy1 contributes to plant resistance against pathogens and, more importantly, RSy1 participates in PeSy1‐induced plant resistance.

## 3 DISCUSSION

Biocontrol microorganisms can colonize the host plants, thereby inhibiting the invasion of plant pathogens and increasing crop yield (Compant et al., 2005; Haggag, 2010). In addition, the PTI response resulting from the sensing of MAMPs by plant PRRs constitutes the line of defence against most pathogens (Boutrot & Zipfel, 2017; Wiesel et al., 2014). However, identification of MAMPs and their recognition receptors is mainly derived from plant pathogens, and there are few reports on the interaction between biocontrol microbial MAMPs and plant targets. Here, we identified a MAMP from a biocontrol actinomycete, PeSy1, which is conserved in rare actinomycetes and is capable of inducing an intense HR in *N*. *benthamiana*. Moreover, the RLCK RSy1 in *N. benthamiana* contributes to PeSy1‐induced plant resistance.

Most secreted proteins are characterized by their small molecular weight and are rich in cysteine residues (Templeton et al., 1994). PeSy1 was screened from our cell death‐like HR analysis of the Hhs.015 secreted proteins (Figure S1). Bioinformatics analysis showed that PeSy1 encodes an 11 kDa protein with four cysteine residues (Table S1), but no domain was identified. Sec and Twin Arginine Translocation (TAT) systems are widely distributed in gram‐positive and gram‐negative bacteria to secrete proteins across the cytoplasmic membrane (Natale et al., 2008). Furthermore, different specialized secretion systems are known in gram‐negative bacteria to perform more specialized functions, including the type I to type VI protein secretion pathways (Costa et al., 2015). On the contrary, gram‐positive bacteria do not have specialized secretion systems, with the exception of the type VII secretion system reported in *Mycobacteria* and other high DNA G+C‐content bacterial species (*Actinobacteria*) (Bottai et al., 2017). Previously, we described *S. yanglingensis* Hhs.015 as a gram‐positive actinomycete with high genomic DNA G+C content (70.94 mol%) (Yan et al., 2012), but we did not predict the type VII system secretion system in Hhs.015 genome. Signal peptide predictionshows that PeSy1 has 24 N‐terminal SP residues and belongs to the standard secretory SP transported by Sec translocation (Figure 2a).

Plant intercellular apoplastic space is a complex place where many important interactions occur, reflecting the close relationship between plants and pathogens (Mott et al., 2014). Using *A. tumefaciens* to mediate the transient expression of PeSy1 in *N. benthamiana*, we found that PeSy1 can induce cell death with or without its SP (Figure 1c). However, PeSy1^ΔSP^ showed HR symptoms later than full‐length PeSy1 (Figure 1c), which is similar to the case of VmE02, a cell death elicitor from *Valsa mali* (Nie et al., 2019). Of note, PeSy1 was observed to be located on the apoplast and cytoplasm/nucleus (Figure 6f), which indicates that it has a variety of ways to play its role. PeSy1 may function in the plant apoplast to induce the immune response in *N. benthamiana*, including a ROS burst, callose deposition, and SA‐ and JA/ET‐mediated resistance pathway activation (Figure 4). In addition, recombinant protein PeSy1‐His treatment also induced resistance in plants against a variety of pathogenic fungi and bacteria (Figure 5). Beneficial microorganisms can induce a systemic defence response, which is controlled by a signal network involving the plant hormones SA and JA/ET (Hammond‐Kosack & Parker, 2003). According to different pathogens, hormone cross‐talk regulates the plant defence response (Koornneef & Pieterse, 2008).

In this research, we found that the plant receptor‐like protein kinase RSy1 is a target of PeSy1 using a pull‐down mass spectrometry screening. The interaction between PeSy1 and RSy1 was confirmed by Co‐IP, BiFC, and MST (Figure 6b,c,e). RSy1 belongs to RLCK in RLK family because it contains only one intracellular kinase domain (Figure S3). Furthermore, PeSy1 and RSy1 showed the same intracellular localization (Figure 6g). The PTI marker genes were up‐regulated after PeSy1 treatment (Figure 7a), indicating that PeSy1 is a MAMP of Hhs.015. Although the known PAMPs interact with the extracellular domain of transmembrane receptors (Dunning et al., 2007; Liu et al., 2012), there are exceptions. The β‐glucan‐binding proteins from Fabaceae plants are thought to be the receptor for β‐glucan, a PAMP of *Phytophthora*, but do not feature the signalling domains found in other innate immune receptors (Mithöfer et al., 2000). Our findings showed that the transcription of *NbPR1* and *NbPR2* was not significantly changed in *RSy1*‐silenced plants after PeSy1 treatment, compared with *GFP*‐silenced plants (Figure 7b). Similarly, silencing of *RSy1* in *N. benthamiana* resulted in susceptibility to *S. sclerotiorum* but still showed disease resistance after PeSy1 treatment (Figure S4). Considering the function redundancy of RSy1, we put forward a hypothesis that there is still an unknown transmembrane receptor to sense apoplastic PeSy1. In this case, as our experiment shows, the activation of immunity depends on the co‐receptors NbBAK1 and NbSOBIR1 (Figure 7d). Subsequently, RSy1 may be recruited by PRR complexes to participate in the transmission of immune signals. More research is needed to verify this hypothesis.

We cannot exclude the possibility that PeSy1 functions as a cytoplasmic effector. Previous studies have shown that necrosis and ethylene‐inducing peptide 1‐like proteins (NLPs) and cerato‐platanin in microorganisms are both PAMPs and effectors (Böhm et al., 2014; Pazzagli et al., 2014). Plant RLCKs are immune regulators that are involved in the recognition and transmission of PAMP signalling in the PTI response (Lin et al., 2013). In previous studies, some RLCKs targeted by phytopathogen effectors also indicated their importance for immunity (Yamaguchi et al., 2013; Zhang et al., 2010). Here, we demonstrate that RSy1 promotes defence‐related gene expression to exert a positive immune regulatory effect (Figure 8d). RSy1 mediates PeSy1‐induced MAMP signalling, as seen by the impaired expression of the PTI marker genes in RSy1‐silenced plants after PeSy1 treatment (Figure 7b). More importantly, overexpression of *RSy1* conferred disease resistance in *N. benthamiana* and treatment with PeSy1 induced stronger *S. sclerotiorum* resistance (Figure 8a,e). Therefore, the interaction between PeSy1 and RSy1 contributes to RLCK signal transmission and rapidly activates the plant disease resistance response. However, it is still unknown how PeSy1 manipulates the function of RSy1 to activate the plant immune response.

Overall, our study reports a novel protein elicitor PeSy1 from *S. yanglingensis* Hhs.015, capable of inducing *N. benthamiana* HR and acting as a MAMP to trigger plant immune responses that enhance resistance against *S. sclerotiorum*, *P. capsica*, and Pst DC3000. Moreover, PeSy1 interacts with RSy1 in *N. benthamiana*. RSy1 participates in PeSy1 signalling to positively regulate PeSy1‐induced plant resistance. Our identification of PeSy1 and its target RSy1 not only contributes to understanding the mechanism of plant immune activation, but also lays the foundation for molecular disease resistance engineering. PeSy1 is expected to become a novel biological pesticide to reduce the harm of diseases and improve the production efficiency of agriculture.

## 4 EXPERIMENTAL PROCEDURES

### 4.1 Strains, plants, and culture conditions

*S. yanglingensis* Hhs.015 was activated on Gause I medium for 7 days. After spore enrichment, mycelial disks were inoculated into a flask with trypticase soy broth medium, then cultured at 220 rpm and 28°C for 48 h. Pst DC3000 was cultured overnight at 28°C in Luria Bertani (LB) broth containing 50 mg/L rifampicin. *S. sclerotiorum* and *P. capsici* were cultured on potato dextrose agar and V8 juice agar, respectively, at 28°C for 72 h. *E. coli* DH5α and BL21 (DE3) were cultured in LB at 220 rpm and 37°C. *A. tumefaciens* GV3101 was cultured in LB with 50 μg/mL rifampicin at 220 rpm and 28°C for 18 h.

*N. benthamiana* and tomato plants were cultivated in a phytotron at 24°C with a 14 h day/10 h night cycle at 75% relative humidity. All strains and *N. benthamiana* seeds were provided by the Laboratory of Integrated Management of Plant Disease in the College of Plant Protection, Northwest A&F University, Shaanxi, Yangling, China. Tomato seeds were purchased from Yonghong Seeds Co., Ltd.

### 4.2 Plasmid construction

Genomic DNA of *S. yanglingensis* Hhs.015 was extracted by a modified CTAB method, and Phanta Max Super‐Fidelity DNA polymerase (Vazyme Biotech) was used for amplification of *PeSy1* (MZ396298.1). According to the one‐step cloning kit, the purified *PeSy1* amplifier was ligated to the potato virus X (PVX) vector that was digested with *Cla*I and *Sal*I for overexpression in plants and screening of protein elicitors. For conducting prokaryotic expression, the *PeSy1* sequence without the SP‐coding sequence was inserted into the pET28a vector between the *Eco*RI and *Sal*I sites to form pET28a:*PeSy1‐His* plasmid. The *RSy1* gene (Niben101Scf02819g03010.1) cloned from a *N. benthamiana* cDNA library and *PeSy1* without terminator codons were cloned into the *Nco*I/*Spe*I digested pCAMBIA1302 vector, resulting in pCAMBIA1302:*RSy1‐GFP* and pCAMBIA1302:*PeSy1‐FLAG* used for carrying out a Co‐IP assay. For BiFC assay, *RSy1* and *PeSy1* were ligated into *Bam*HI/*Sal*I‐digested pSPYNE(R)173 and *Bam*HI/*Cla*I‐digested pSPYCE(M) to generate the pSPYNE(R)173‐*RSy1* and pSPYCE(M)‐*PeSy1* vectors, respectively. To create the expression constructs used for the MST assay, the *RSy1* amplification product was ligated to pGEX‐6p‐1 vector digested with *Bam*HI and *Eco*RI. To create constructs for subcellular localization analysis, the *PeSy1* and *PeSy1* ^ΔSP^ fragment was ligated into digested pCAMBIA1302, resulting in pCAMBIA1302:*PeSy1‐GFP* and pCAMBIA1302:*PeSy* ^ΔSP^ *‐GFP*. The *PeSy1* fragment was cloned into *Cla*I/*Spe*I‐digested pICH86988 vector to form pICH86988:*PeSy1‐mCherry*.The constructs used for VIGS in *N. benthamiana* were generated in the TRV2 vector (Liu et al., 2002). All constructs were validated by sequencing by Tsingke Biotech (Beijing, China). All primers used are described in Table S1.

### 4.3 Trypan blue staining and detection of electrical conductivity in *N. benthamiana* leaves

For agroinfiltration in *N. benthamiana* leaves, competent *A. tumefaciens* GV3101 was transformed with PVX‐*PeSy1* recombinant vector by electroporation. To observe the HR clearly, the leaves of transient expression were stained with trypan blue according to Qi's method with a slight modification (Qi et al., 2016). The whole leaves were soaked in 1 mg/mL trypan blue dye solution (trypan blue dissolved in equal proportions solution of water, glycerol, lactic acid, and water‐saturated phenol) and boiled for 3 min. After standing in the dark for 14 h, the leaves were decolourized with 2.5 g/mL chloral hydrate. The degree of leaf cell death can be characterized by ion leakage (Mittler et al., 1999). After agroinfiltration in *N. benthamiana* leaves for 4 days, six leaf disks (1 cm diameter) were put into a 5‐mL centrifuge tube with 4 mL of double‐distilled water and left to sit overnight. A conductivity meter (Mettler‐Toledo) was used to measure the liquid conductivity (E1) after shaking. Then the disks were boiled in sealed tubes for 25 min and the conductivity (E2) measured again after cooling. The conductivity ratio was calculated as (E1/E2) × 100.

### 4.4 Bioinformatics analysis

With regard to the analysis of Hhs.015 secretory proteins, the signal peptides of the proteome were predicted using SignalP (http://www.cbs.dtu.dk/services/SignalP/) and proteins with transmembrane helix structure were excluded using TMHMM (http://www.cbs.dtu.dk/services/TMHMM/). The conserved domains were searched with SMART (http://smart.embl‐heidelberg.de/). The amino acid composition, molecular weight, and isoelectric point of proteins were analysed by Expasy (https://web.expasy.org/protparam/) (Duvaud et al., 2021). The tertiary structure of protein was predicted by Phyre^2^ (http://www.sbg.bio.ic.ac.uk/phyre2/) and visualized by PymolWin (Kelley et al., 2015). The homologous sequences were compared using BLASTP in the NCBI database, and 20 sequences with high similarity were selected. Phylogenetic dendrograms were constructed using MEGA with the neighbour‐joining method. DNAMAN was used to analyse the sequence conservation. The PeSy1 data were submitted to the NCBI repository and can be found under GenBank accession number MZ396298.1.

### 4.5 Prokaryotic expression and purification of PeSy1

The recombinant vector pET28a:*PeSy1‐His* was transferred into competent *E. coli* BL21 (DE3) cells by heat shock. The *E. coli* BL21 (DE3) cells were induced with 0.2 mM IPTG at 180 rpm and 16°C for 20 h. For protein extraction, the induced culture cells were harvested by centrifugation at 5000 × *g* and 4°C for 5 min, then washed three times in phosphate‐buffered saline (PBS; 20 mM Na_2_HPO_4_, 300 mM NaCl, pH 8.0) with 0.02 mg/mL lysozyme, then 1 mM phenylmethanesulfonyl fluoride (PMSF) was added and reacted at 25°C for 10 min. After disruption using an ultrasonic disruptor (Scientz), the supernatant containing crude protein was mixed with the same volume of equlibration buffer and filtered through a 0.22‐μm filter (Millipore). The filtrate was purified by affinity chromatography using HisPur Ni‐NTA resin (Thermo Fisher Scientific) according to the manufacturer's instructions, and the concentration of PeSy1 was measured by NanoDrop spectrophotometer (Thermo Fisher Scientific). The purified protein with concentration higher than 0.1 mg/ml was dialysed against PBS for subsequent experiments.

### 4.6 Detection of immune response in *N. benthamiana*

To explore the minimum concentration of *N. benthamiana* cell death caused by PeSy1, the leaves were injected with PeSy1 pure protein at concentrations of 5, 10, 20, 40, 60, and 80 μM. The PeSy1 pure protein of appropriate concentration was injected into *N. benthamiana* to observe its immune response. Leaves at 24 h posttreatment (hpt) were cut into small pieces, 1 × 1 cm. H_2_O_2_ was detected by DAB staining and callose was detected by aniline blue staining as described previously (Niu et al., 2011). H_2_O_2_ production was examined under a light microscope and the callose deposition observed using a fluorescence microscope.

### 4.7 RNA extraction and RT‐qPCR analysis

A Quick RNA isolation Kit (Huayueyang) was used to extract the total RNA from *N. benthamiana* leaves following the manufacturer's instructions. One microgram of total RNA was measured by a NanoDrop spectrophotometer and reverse transcribed into cDNA using the RevertAid First Strand cDNA Synthesis Kit (Thermo Fisher Scientific). Quantitative PCR was performed using a LightCycler 96 System (Roche) with 2 × RealStar Green Power Mixture (GenStar) to determine the gene expression. All the experiments were performed in three biological replicates, and each experiment consisted of three technical replicates. The *Actin* gene in *N. benthamiana* was used as a reference control for normalization. The relative expression of each gene was calculated using the comparative 2^−∆∆*C*t^ method (Livak & Schmittgen, 2001). All primers used for the RT‐qPCR are described in Table S2.

### 4.8 Bioassay for PeSy1‐induced disease resistance in plants

*N. benthamiana* leaves were treated with 5 μM PeSy1 or PBS as a negative control, and intact leaf sections were collected 12 h later for pathogen inoculation. The cultured plant pathogens, *S. sclerotiorum* and *P. capsici*, were inoculated on the leaves. The lesion size of 48 hpi was calculated by ImageJ software. Additionally, an equal volume of 10 μM PeSy1‐His or PBS was sprayed on 5‐ to 6‐week‐old tomato leaves and maintained in wet conditions at 24°C for 24 h. The harvested Pst DC3000 cells were suspended in 10 mM MgCl_2_ containing 0.04% Silwet 77 at optical density (OD)_600_ 1.2 and then sprayed evenly on leaves. After infection for 48 h, the leaves were ground into powder and a dilution series of sample suspensions was spread on King's B medium containing 50 μg/mL rifampicin to determine the colony‐forming units (cfu). The experiment was repeated with at least three biological replicates.

### 4.9 Pull‐down and mass spectrometry analysis

The total protein from *N. benthamiana* leaves was extracted using native lysis buffer (50 mM Tris, 150 mM NaCl, 1 mM EDTA, 5% glycerol) (Solaribio) containing 1 mM PMSF, and 1% proteinase inhibitor cocktail following the manufacturer's instructions. PeSy1‐His and total protein were first incubated for 10 min at room temperature, and then left at 4°C for 50 min. The interaction targets were enriched by pull down using His‐tag magnetic agarose beads (Beaver) and protein samples were boiled for 10 min in 5× SDS loading buffer for SDS‐PAGE. After treatment by in‐gel digestion, interaction targets were identified using a Q Exactive HF‐X mass spectrometer (Thermo Fisher Scientific).

### 4.10 Co‐IP assay

*A. tumefaciens* cells carrying pCAMBIA1302:*RSy1‐GFP*, pCAMBIA‐1302:*PeSy1*‐*FLAG*, and P19 were co‐injected into *N. benthamiana* leaves and the total protein was extracted using native lysis buffer at 60 hpa as described above. The lysed protein sample was used as the input sample and the remaining *N. benthamiana* proteins were incubated with anti‐FLAG magnetic beads (Beaver). Buffer (20 mM Tris, 150 mM glycine, pH 7.5) was used for washing three times. To avoid nonspecific band contamination in the results, 0.1 M glycine‐HCl (pH 2.8) in 1 M Tris (pH 9.0) was used for acid elution. Anti‐GFP (Abmart) and anti‐FLAG (Abmart) monoclonal antibody were detected by western blotting.

### 4.11 BiFC assay

pSPYNE(R)173‐RSy1 and pSPYCE(M)‐PeSy1 vectors were transformed into *A. tumefaciens* and co‐infiltrated into *N. benthamiana* leaves. Empty vector was used as the negative control. Two days after agroinfiltration, yellow fluorescent protein fluorescence was observed using an Olympus FV3000 confocal laser microscope. All the assays were repeated at least three times.

### 4.12 Microscale thermophoresis assay

PeSy1‐His was labelled using a protein labelling kit RED (Nanotemper Technologies) following the manufacturer's instructions and incubated with PBST (PBS with 0.05% Tween 20) before 30 min at room temperature. The labelled proteins were individually reacted with serially diluted RSy1‐GST in 16 standard treated capillaries. The measurements were performed using a Monolith NT.115 (Nanotemper Technologies). The information of sample name and concentration was set by the Monolith Control system. The excitation power was set to 40% and the MST power was selected as medium. The experiment was carried out with three times biological repetition. The data were analysed by the Monolith Affinity Analysis program.

### 4.13 Subcellular localization analysis

*N. benthamiana* leaves were imaged 2 days after agroinfiltration with pCAMBIA1302 or pICH86988 constructs using a laser confocal microscope (Olympus). GFP fluorescence was observed with an excitation wavelength of 488 nm and mCherry fluorescence was observed with an excitation wavelength of 561 nm. To analyse the fluorescence distribution after plasmolysis, cells were treated with 1.5 M NaCl for 5 min. *A. tumefaciens* infiltration carrying the pCAMBIA1302 or pICH86988 empty vector was used as a control.

### 4.14 VIGS in *N. benthamiana*

*N. benthamiana* leaves at the four‐leaf stage were infiltrated with cultured *A. tumefaciens* GV3101 containing a mixture of TRV1, P19, and TRV2 constructs (OD_600_ = 0.4). TRV2:*GFP* and TRV2:*PDS* (phytoene desaturase) were used as the control groups. Three weeks after agroinfiltration, the albino phenotype caused by *PDS* gene silencing was used as a reference (Ma et al., 2015), and the silencing efficiency of the target genes was determined by RT‐qPCR at the position corresponding to the processed leaves. The primers used for RT‐qPCR are listed in Table S2. All experiments were performed with at least three biological replicates.

### 4.15 Statistical analysis

The data were reported as mean ± *SE* from three independent biological replicates and analysed with GraphPad Prism 8 software. Significant differences between mean values were evaluated using Student's *t* test (*p* < 0.05).

## CONFLICT OF INTEREST STATEMENT

The authors declare that they have no conflict of interest.

## Supporting information


Figure S1 Transient expression of predicted secreted protein in tobacco leads to cell death. *Agrobacterium* carrying five recombinant vectors of Hhs.015_GM7245, Hhs.015_GM2882, Hhs.015_GM7061,

Figure S2 Protein interactions of candidate *Nicotiana benthamiana* target proteins (Niben101Scf09075g03002.1, Niben101Scf02819g03010.1, Niben101Scf11721g00002.1, Niben101Scf04386g04007.1) and PeSy1‐FLAG were determined by co‐immunoprecipitation assay. Anti‐FLAG and anti‐GFP were used to detect protein expression. The red asterisk indicates the band of the target protein. Molecular mass markers (kDa) are shown on the left.

Figure S3 Schematic drawings of the structural domain of RSy1. The coloured box shows the serine/threonine kinase domain.

Figure S4 Silencing of *RSy1* did not affect PeSy1‐induced *Nicotiana benthamiana* resistance against *Sclerotinia sclerotiorum*. (a) Representative leaves showing disease lesions of *GFP‐* or *RSy1*‐silenced *N. benthamiana* infected with *S. sclerotiorum*. The leaves (*n* = 5) treated with 5 μM PeSy1‐His or phosphate‐buffered saline (PBS) control were inoculated with *S. sclerotiorum* and photographed 36 h postinoculation (hpi). Bars are 1 cm. (e) Lesion area was measured 36 hpi. (b) Representative images of TRV2‐*RSy1* plants compared to TRV2‐*GFP* controls. And no apparent developmental phenotype was observed in TRV2‐*RSy1* plants. (c) The RSy1 expression levels in silenced plants were analysed by reverse transcription‐quantitative PCR. (d) Transcript accumulation of *NbPR1* and *NbPR4* in TRV2‐*RSy1* plants. *NbActin* was used as the internal reference gene to standardize the samples. The TRV2‐*GFP* were used as control group. Bars indicate ± *SE*. The statistical analyses were performed with Student’s *t* test. NS, no significant difference; **p <0.01, ***p < 0.001. These experiments were repeated three times with similar results.

Table S1 Candidate protein elicitors and basic characteristics.

Table S2 Primers used for PCR in this study.

Table S3 Primers used for reverse transcription‐quantitative PCR in this study.

## Data Availability

The data that support the findings of this study are available from the corresponding author upon reasonable request.
