# Supplementary material for: A novel protein elicitor (PeSy1) from Saccharothrix yanglingensis induces plant resistance and interacts with a receptor‐like cytoplasmic kinase in Nicotiana benthamiana
Source: Mol Plant Pathol. 2023 Mar 5;24(5):436–51. doi: 10.1111/mpp.13312 (PMC10098051; doi:10.1111/mpp.13312)
Supplement: Supplementary file 1 — Figure S1 Transient expression of predicted secreted protein in tobacco leads to cell death. Agrobacterium carrying five recombinant vectors of Hhs.015_GM7245, Hhs.015_GM2882, Hhs.015_GM7061, [file MPP-24-436-s003.docx]

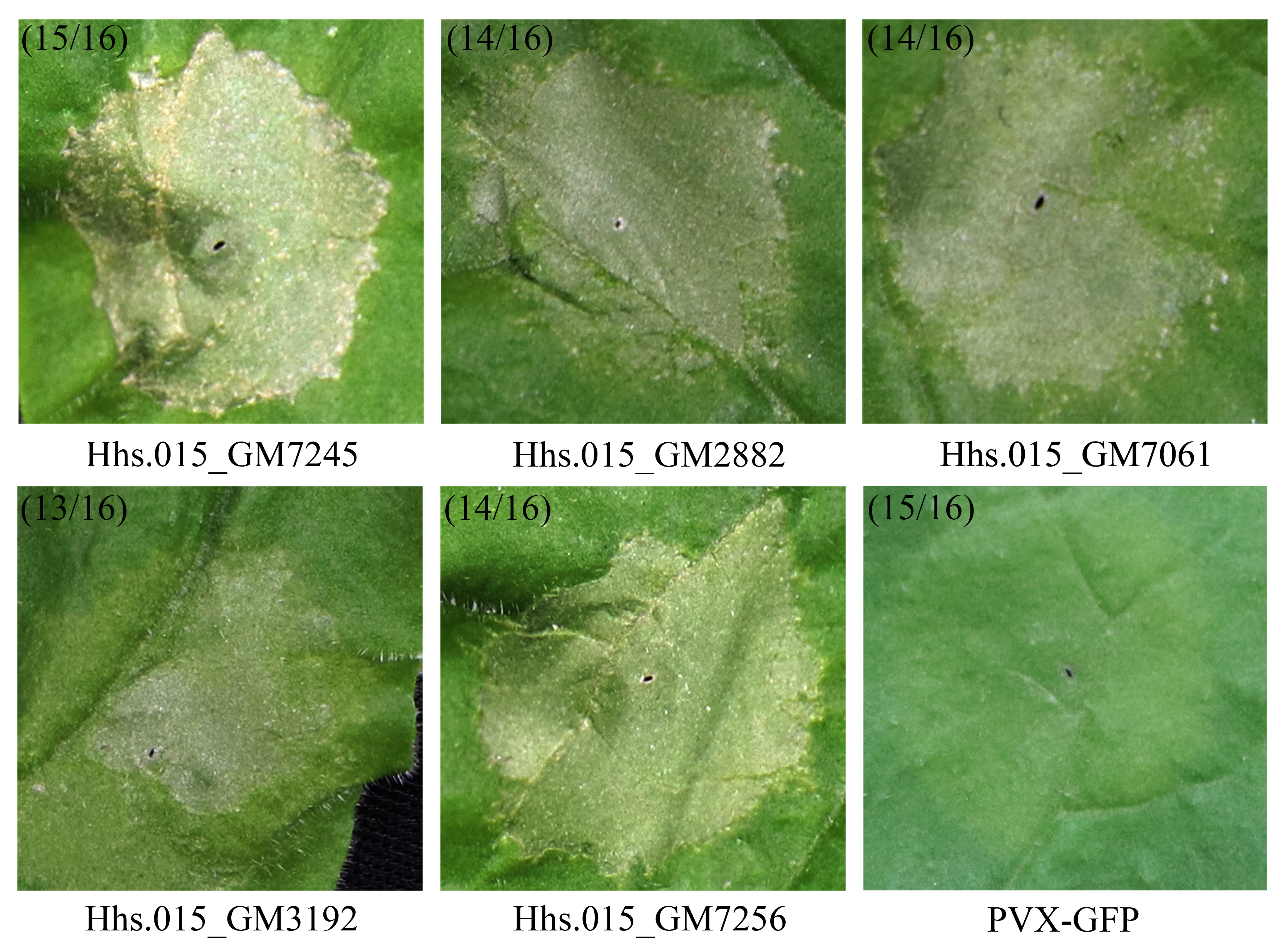


**FIGURE** **S1** Transient expression of predicted secreted protein in tobacco leads to cell death. *Agrobacterium* carrying five recombinant vectors of Hhs.015_GM7245, Hhs.015_GM2882, Hhs.015_GM7061, Hhs.015_GM3192 and Hhs.015_GM7256 showed obvious cell death in tobacco after transient expression for 3-6 days. PVX-GFP was injected as a negative control. In the figure, n/16 means a total of 16 repetitions, and the same result appears n times.
