# Supplementary material for: A novel protein elicitor (PeSy1) from Saccharothrix yanglingensis induces plant resistance and interacts with a receptor‐like cytoplasmic kinase in Nicotiana benthamiana
Source: Mol Plant Pathol. 2023 Mar 5;24(5):436–51. doi: 10.1111/mpp.13312 (PMC10098051; doi:10.1111/mpp.13312)
Supplement: Supplementary file 2 — Figure S2 Protein interactions of candidate Nicotiana benthamiana target proteins (Niben101Scf09075g03002.1, Niben101Scf02819g03010.1, Niben101Scf11721g00002.1, Niben101Scf04386g04007.1) and PeSy1‐FLAG were determined by co‐immunoprecipitation assay. Anti‐FLAG and anti‐GFP were used to detect protein expression. The red asterisk indicates the band of the target protein. Molecular mass markers (kDa) are shown on the left. [file MPP-24-436-s001.docx]

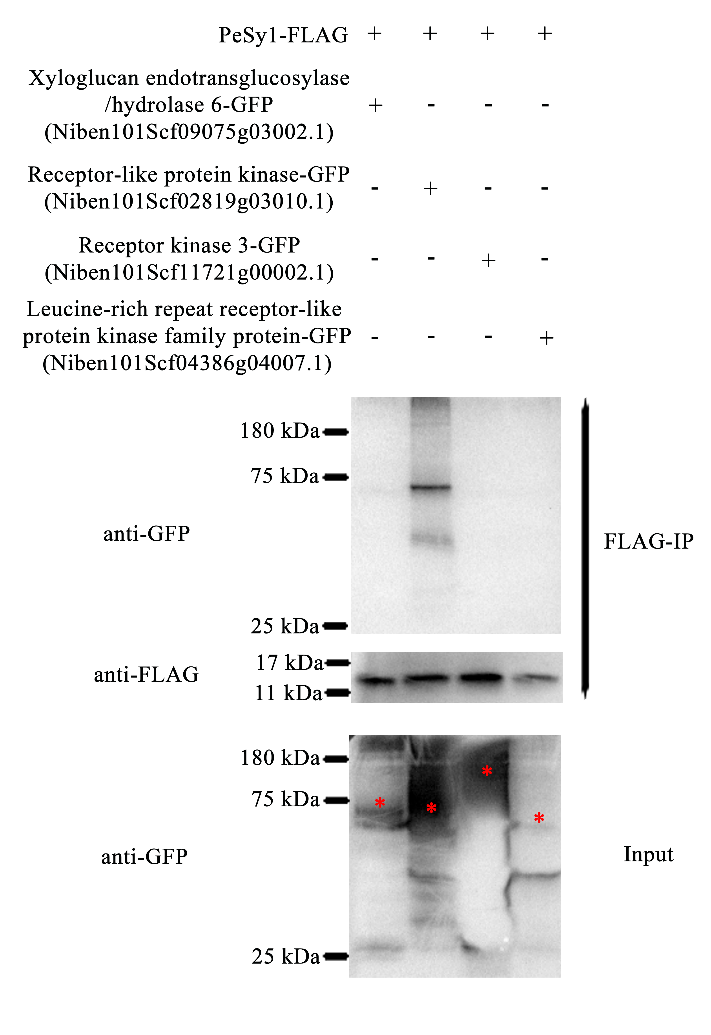


**FIGURE S2** Protein interactions of candidate tobacco target proteins (Niben101Scf09075g03002.1, Niben101Scf02819g03010.1, Niben101Scf11721g00002.1, Niben101Scf04386g04007.1) and PeSy1-FLAG were determined by Co-IP assay. Anti-FLAG and anti-GFP were used to detect protein expression. The red asterisk indicates the band of the target protein. Molecular mass markers (kilodalton) are shown on the left.
