# Supplementary material for: A novel protein elicitor (PeSy1) from Saccharothrix yanglingensis induces plant resistance and interacts with a receptor‐like cytoplasmic kinase in Nicotiana benthamiana
Source: Mol Plant Pathol. 2023 Mar 5;24(5):436–51. doi: 10.1111/mpp.13312 (PMC10098051; doi:10.1111/mpp.13312)
Supplement: Supplementary file 3 — Figure S3 Schematic drawings of the structural domain of RSy1. The coloured box shows the serine/threonine kinase domain. [file MPP-24-436-s006.docx]

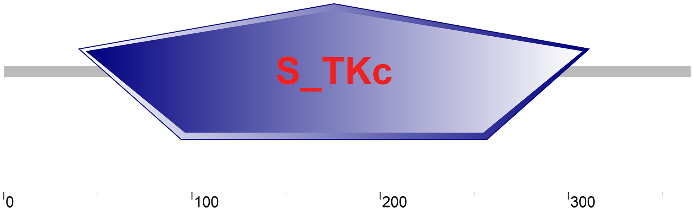


**FIGURE** **S3** Schematic drawings of the structural domain of RSy1. The coloured box shows the serine/threonine kinase domain.
