# Supplementary material for: A novel protein elicitor (PeSy1) from Saccharothrix yanglingensis induces plant resistance and interacts with a receptor‐like cytoplasmic kinase in Nicotiana benthamiana
Source: Mol Plant Pathol. 2023 Mar 5;24(5):436–51. doi: 10.1111/mpp.13312 (PMC10098051; doi:10.1111/mpp.13312)

**FIGURE** **S4** Silencing of *RSy1* did not affect PeSy1-induced *N. benthamiana* resistance against *S. sclerotiorum*. (a) Representative leaves showing disease lesions of *GFP-* or *RSy1-*silenced *N. benthamiana* infected with *S. sclerotiorum*. The leaves (n = 5) treated with 5 μM PeSy1-His or PBS buffer control were inoculated with *S. sclerotiorum* and photographed 36 h post-inoculation (hpi). Bars are 1 cm. (e) Lesion area was measured 36 hpi. (b) Representative images of TRV2-*RSy1* plants compared to TRV2-*GFP* controls. And no apparent developmental phenotype was observed in TRV2-*RSy1* plants. (c) The *RSy1* expression levels in silenced plants were analyzed by qRT-PCR. (d) Transcript accumulation of *NbPR1* and *NbPR4* in TRV2-*RSy1* plants. *NbActin* was used as the internal reference gene to standardize the samples. The TRV2-*GFP* were used as control group. Bars indicate±SE. The statistical analyses were performed with student’s t-test. NS, No significant difference; **, *P* < 0.01; ***, *P* < 0.001. These experiments were repeated three times with similar results.


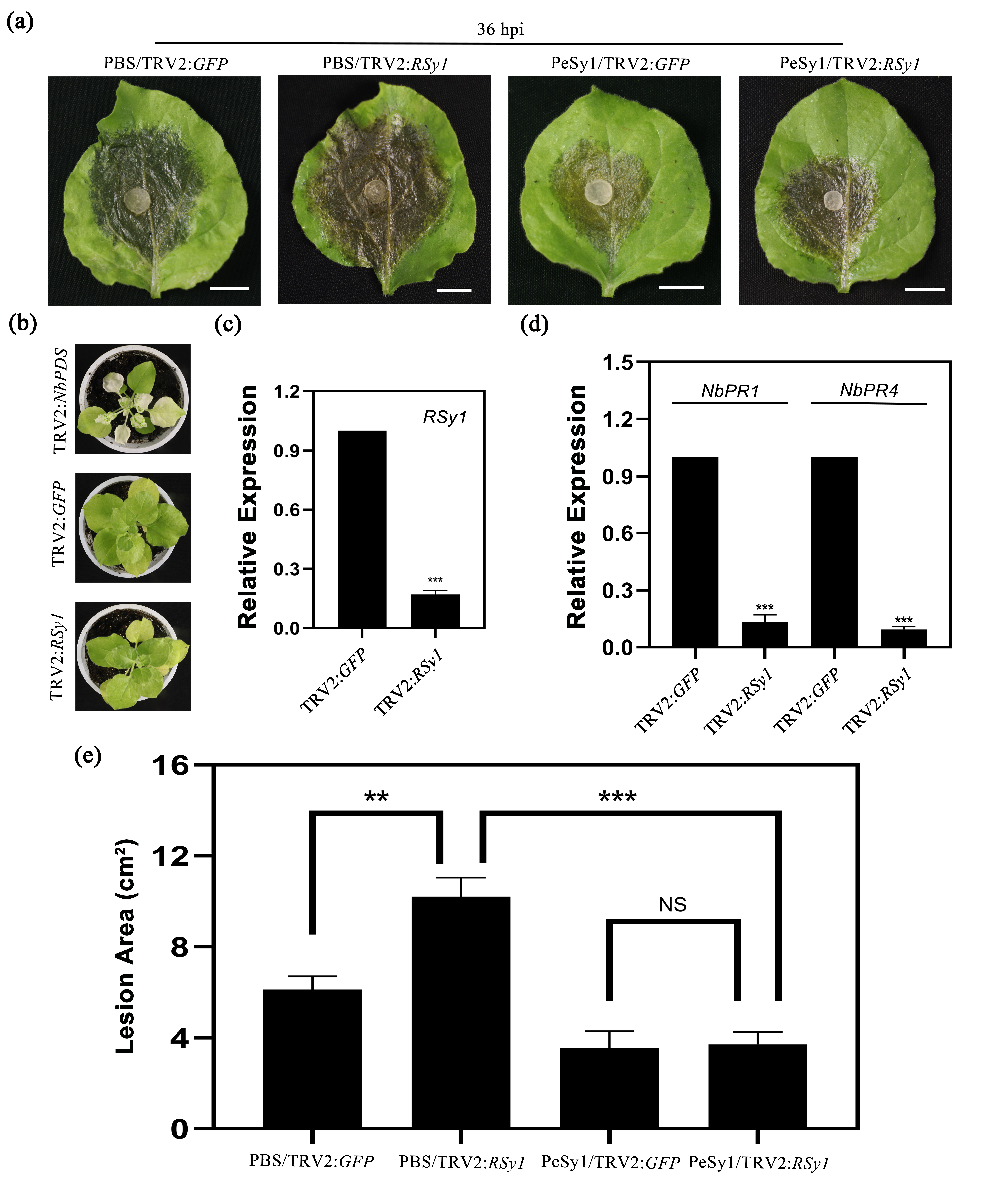

Supplement: Supplementary file 4 — Figure S4 Silencing of RSy1 did not affect PeSy1‐induced Nicotiana benthamiana resistance against Sclerotinia sclerotiorum. (a) Representative leaves showing disease lesions of GFP‐ or RSy1‐silenced N. benthamiana infected with S. sclerotiorum. The leaves (n = 5) treated with 5 μM PeSy1‐His or phosphate‐buffered saline (PBS) control were inoculated with S. sclerotiorum and photographed 36 h postinoculation (hpi). Bars are 1 cm. (e) Lesion area was measured 36 hpi. (b) Representative images of TRV2‐RSy1 plants compared to TRV2‐GFP controls. And no apparent developmental phenotype was observed in TRV2‐RSy1 plants. (c) The RSy1 expression levels in silenced plants were analysed by reverse transcription‐quantitative PCR. (d) Transcript accumulation of NbPR1 and NbPR4 in TRV2‐RSy1 plants. NbActin was used as the internal reference gene to standardize the samples. The TRV2‐GFP were used as control group. Bars indicate ± SE. The statistical analyses were performed with Student’s t test. NS, no significant difference; **p <0.01, ***p < 0.001. These experiments were repeated three times with similar results. [file MPP-24-436-s004.docx]
