# Supplementary material for: A novel protein elicitor (PeSy1) from Saccharothrix yanglingensis induces plant resistance and interacts with a receptor‐like cytoplasmic kinase in Nicotiana benthamiana
Source: Mol Plant Pathol. 2023 Mar 5;24(5):436–51. doi: 10.1111/mpp.13312 (PMC10098051; doi:10.1111/mpp.13312)
Supplement: Supplementary file 5 — Table S1 Candidate protein elicitors and basic characteristics. [file MPP-24-436-s002.docx]

**Table S1** Candidate protein elicitors and basic characteristics

| Gene Id | Number of Cys/Total Length (aa) | Signal peptide (aa) | Gene Id | Number of Cys/Total Length (aa) | Signal peptide (aa) |
| --- | --- | --- | --- | --- | --- |
| Hhs.015_GM0262 | 3/313 | 1-27 | Hhs.015_GM3590 | 2/128 | 1-16 |
| Hhs.015_GM0424 | 3/143 | 1-25 | Hhs.015_GM4017 | 2/68 | 1-23 |
| Hhs.015_GM1200 | 1/206 | 1-27 | Hhs.015_GM4175 | 0/166 | 1-20 |
| Hhs.015_GM1357 | 4/116 | 1-20 | Hhs.015_GM4381 | 5/331 | 1-23 |
| Hhs.015_GM1516 | 2/137 | 1-15 | Hhs.015_GM4398 | 6/449 | 1-22 |
| Hhs.015_GM1553 | 2/59 | 1-18 | Hhs.015_GM4438 | 4/171 | 1-22 |
| Hhs.015_GM1974 | 2/322 | 1-21 | Hhs.015_GM4667 | 4/237 | 1-23 |
| Hhs.015_GM1982 | 1/370 | 1-22 | Hhs.015_GM4932 | 4/126 | 1-27 |
| Hhs.015_GM2440 | 3/334 | 1-30 | Hhs.015_GM5027 | 3/279 | 1-21 |
| Hhs.015_GM2374 | 9/325 | 1-36 | Hhs.015_GM5260 | 1/190 | 1-18 |
| Hhs.015_GM2825 | 6/421 | 1-16 | Hhs.015_GM5262 | 8/351 | 1-17 |
| Hhs.015_GM2882 | 5/152 | 1-19 | Hhs.015_GM6391 | 2/430 | 1-20 |
| Hhs.015_GM3192 | 0/455 | 1-27 | Hhs.015_GM7061 | 2/179 | 1-19 |
| Hhs.015_GM3229 | 0/246 | 1-25 | Hhs.015_GM7148 | 3/180 | 1-16 |
| Hhs.015_GM3238 | 1/501 | 1-21 | Hhs.015_GM7245 | 4/109 | 1-24 |
| Hhs.015_GM3335 | 4/190 | 1-22 | Hhs.015_GM7256 | 8/340 | 1-24 |
