# Supplementary material for: A novel protein elicitor (PeSy1) from Saccharothrix yanglingensis induces plant resistance and interacts with a receptor‐like cytoplasmic kinase in Nicotiana benthamiana
Source: Mol Plant Pathol. 2023 Mar 5;24(5):436–51. doi: 10.1111/mpp.13312 (PMC10098051; doi:10.1111/mpp.13312)
Supplement: Supplementary file 6 — Table S2 Primers used for PCR in this study. [file MPP-24-436-s005.docx]

**Table S2** Primers used for PCR in this study

| **Primer name** | **Sequence (5′to 3′)** | **Purpose** |
| --- | --- | --- |
| PVX:PeSy1-F | GCACCAGCTAGCatcgat  ATGGTGAGACGAACCCTGACCGC | Clone PeSy1 to PVX for expression in *N. benthamiana* |
| PVX:PeSy1^ΔSP^ -F | GCACCAGCTAGCatcgat  ATGGTCACGTCGGTCGACGTGC |  |
| PVX:PeSy1-R | CGTTCATCGGCGgtcgac  TCACGGCCAGGGGCCGC |  |
| pET28a:PeSy1-HIS-F | CAAATGGGTCGCGGATCCGAATtc  ATGGTCACGTCGGTCGACGTGC | Clone PeSy1 to pET28a for expression in *E. coli* |
| pET28a:PeSy1-HIS-R | GAGTGCGGCCGCAAGCTTGTCGac  CGGCCAGGGGCCGCTGTT |  |
| pCAMBIA1302:PeSy1 -F | GGGGACTCTTGACCATGGta  ATGGTGAGACGAACCCTGACCGC | Clone PeSy1 to pCAMBIA1302 for expression in *N. benthamiana* |
| pCAMBIA1302:PeSy1-FLAG-R | CTCACCATCCTAGGACTAgt  TTACTTATCGTCGTCATCCTT GTAatcCGGCCAGGGGCCGCTGTT |  |
| pCAMBIA-1302:PeSy1-GFP-R | CTCACCATCCTAGGACTAgt  CGGCCAGGGGCCGCTGTT |  |
| pCAMBIA1302:RSy1-GFP-F | GGGGACTCTTGACCATGGta  ATGGCTTTTTCGTCTATATTTTG | Clone RSy1 to pCAMBIA1302 for expression in *N. benthamiana* |
| pCAMBIA1302:RSy1-GFP-R | CTCACCATCCTAGGACTAgt  TACCTCTACCTTTTCAATTTCTG |  |
| pICH86988:PeSy1 -F | CATTTACAATTATCGat  ATGGTGAGACGAACCCTGACCGC | Clone PeSy1 to pICH86988 for expression in *N. benthamiana* |
| pICH86988:PeSy1-mCherry-R | CTCACCCTAGGACTAgt  CGGCCAGGGGCCGCTGTT |  |
| pSPYNE(R)173-RSy1-F | CCCAGGCCTACTACTAGTGGatcc  ATGGCTTTTTCGTCTATATTTTG | Clone RSy1 to pSPYNE(R)173 for expression in *N. benthamiana* |
| pSPYNE(R)173-RSy1-R | AGCGGTACCCTCGAGGTcgac  TACCTCTACCTTTTCAATTTCTG |  |
| pSPYCE(M)-PeSy1-F | TGGCGCGCCACTAGTGGAtcc  ATGGTCACGTCGGTCGACGTGC | Clone PeSy1 to pSPYCE(M) for expression in *N. benthamiana* |
| pSPYCE(M)-PeSy1-R | GAGGTCGACAGTACTATCgat  CGGCCAGGGGCCGCTGTT |  |
| pGEX-6p-1:RSy1-GST-F | CCAGGGGCCCCTGGGAtcc  ATGGCTTTTTCGTCTATATTTTG | Clone RSy1 to pGEX-6p-1 for expression in *E. coli* |
| pGEX-6p-1:RSy1-GST-R | GAGTCGACCCGGGAAttc  TACCTCTACCTTTTCAATTTCTG |  |
| TRV2:RSy1-F | GTGAGCTCGGTACCGGATcc  TGCAATGTTAGGGAAGGCATC | Clone RSy1 to TRV2 for expression in *N. benthamiana* |
| TRV2:RSy1-R | TGAGTAAGGTTACCGAATtc  CCTTTCAGTAGCTCAACAACCTCA |  |
