# Supplementary material for: A novel protein elicitor (PeSy1) from Saccharothrix yanglingensis induces plant resistance and interacts with a receptor‐like cytoplasmic kinase in Nicotiana benthamiana
Source: Mol Plant Pathol. 2023 Mar 5;24(5):436–51. doi: 10.1111/mpp.13312 (PMC10098051; doi:10.1111/mpp.13312)
Supplement: Supplementary file 7 — Table S3 Primers used for reverse transcription‐quantitative PCR in this study. [file MPP-24-436-s007.docx]

**Table S3 Primers used for qRT-PCR in this study**

| **Primer name** | **Sequence (5′to 3′)** |
| --- | --- |
| qRT-NbActin-F | TGGTCGTACCACCGGTATTGTGTT |
| qRT-NbActin-R | TCACTTGCCCATCAGGAAGCTCAT |
| qRT-NbPR1-F | CCGCCTTCCCTCAACTCAAC |
| qRT-NbPR1-R | GCACAACCAAGACGTACTGAG |
| qRT-NbPR2-F | AGGTGTTTGCTATGGAATGC |
| qRT-NbPR2-R | TCTGTACCCACCATCTTGC |
| qRT-NbPR4-F | GGCCAAGATTCCTGTGGTAGAT |
| qRT-NbPR4-R | CACTGTTGTTTGAGTTCCTGTTCCT |
| qRT-NbERF1-F | GCTCTTAACGTCGGATGGTC |
| qRT-NbERF1-R | AGCCAAACCCTAGCTCCATT |
| qRT-NbCYP71D20-F | AAGGTCCACCGCACCATGTCCTTAGAG |
| qRT-NbCYP71D20-R | AAGAATTCCTTGCCCCTTGAGTACTTGC |
| qRT-NbPTI5-F | CCTCCAAGTTTGAGCTCGGATAGT |
| qRT-NbPTI5-R | CCAAGAAATTCTCCATGCACTCTGTC |
| qRT-NbACRE31-F | AATTCGGCCATCGTGATCTTGGTC |
| qRT-NbACRE31-R | GAGAAACTGGGATTGCCTGAAGGA |
| qRT-NbWRKY7-F | CACAAGGGTACAAACAACACAG |
| qRT-NbWRKY7-R | GGTTGCATTTGGTTCATGTAAG |
| qRT-NbWRKY8-F | AACAATGGTGCCAATAATGC |
| qRT-NbWRKY8-R | TGCATATCCTGAGAAACCATT |
| qRT-RSy1-F | CTTCTTGATTGGAAGCGACG |
| qRT-RSy1-R | CAGCAACTTGGGCTTGGAA |
| qRT-NbPDF1.2-F | ATCTGTCTGGGGAAATGGCA |
| qRT-NbPDF1.2-R | CATGGTCCCTTGAAACGGTG |
| qRT-COI1-F | AACTGGTCGGGATCTCTTGG |
| qRT-COI1-R | TAGGCAAGTATATGGGCGGG |
| qRT-NbBAK1-F | CTAGATTGGGTCAAGGGACTTC |
| qRT-NbBAK1-R | GCTGTTCCACCTCTTCTTCTT |
| qRT-NbSOBIR1-F | GCAGAATTGGATCAGCACTTC |
| qRT-NbSOBIR1-R | CTTCCTGTTACTATGTTTATGG |
